# Cell geometry determines symmetric and asymmetric division plane selection in *Arabidopsis* early embryos

**DOI:** 10.1371/journal.pcbi.1006771

**Published:** 2019-02-11

**Authors:** Julien Moukhtar, Alain Trubuil, Katia Belcram, David Legland, Zhor Khadir, Aurélie Urbain, Jean-Christophe Palauqui, Philippe Andrey

**Affiliations:** 1 Institut Jean-Pierre Bourgin, INRA, AgroParisTech, CNRS, Université Paris-Saclay, 78000 Versailles, France; 2 MaIAGE, INRA, Université Paris-Saclay, 78350, Jouy-en-Josas, France; 3 INRA, UMR782 Génie et Microbiologie des Procédés Alimentaires, 78850 Thiverval-Grignon, France; Oxford, UNITED KINGDOM

## Abstract

Plant tissue architecture and organ morphogenesis rely on the proper orientation of cell divisions. Previous attempts to predict division planes from cell geometry in plants mostly focused on 2D symmetric divisions. Using the stereotyped division patterns of *Arabidopsis thaliana* early embryogenesis, we investigated geometrical principles underlying plane selection in symmetric and in asymmetric divisions within complex 3D cell shapes. Introducing a 3D computational model of cell division, we show that area minimization constrained on passing through the cell centroid predicts observed divisions. Our results suggest that the positioning of division planes ensues from cell geometry and gives rise to spatially organized cell types with stereotyped shapes, thus underlining the role of self-organization in the developing architecture of the embryo. Our data further suggested the rule could be interpreted as surface minimization constrained by the nucleus position, which was validated using live imaging of cell divisions in the stomatal cell lineage.

## Author summary

The proper positioning of division planes is key for correct development and morphogenesis of organs, in particular in plants were cellular walls prevent cell rearrangements. Elucidating how division planes are selected is therefore essential to decipher the cellular bases of plant morphogenesis. Previous attempts to identify geometrical rules relating cell shape and division plane positioning in plants mostly focused on symmetric divisions in tissues reduced to 2D geometries. Here, we combined 3D quantitative image analysis and a new 3D cell division model to evaluate the existence of geometrical rules in asymmetrical and symmetrical divisions of complex cell shapes. We show that in the early embryo of the model plant *Arabidopsis thaliana*, which presents stereotyped but complex cell division patterns, a single geometrical rule (area minimization constrained on passing through the cell centroid) recapitulates the complete sequence of division events. This new rule, valid for both symmetrical and asymmetrical divisions, generalizes previously proposed cell division rules and can be interpreted based on the dynamics of the cytoskeleton and on the positioning of the nucleus, a hypothesis that we validated using leaf cell division patterns. This work highlights the importance of self-organization in plant early morphogenesis and the emergence of robust cellular organizations based on geometrical feedback loops between cell geometry and division plane selection.

## Introduction

In plants, patterns of cell divisions combined with growth and differentiation determine the hierarchical organization into tissues and organs. Cells can proliferate via symmetric divisions while asymmetric divisions are mainly associated with initiation of new cell types, layers and developmental patterns [1, 2]. In asymmetric division, cleavage plane formation has often been associated with cell polarity. A classical paradigm is that cleavage plane position would be driven by some asymmetrically distributed cell fate determinant, which would drive daughter cells towards different developmental fates. An alternative view is that daughter cells would share initially the same determinant but would be subjected to different positional cues causing them to develop differently [3]. Hence, elucidating the principles that govern symmetric and asymmetric divisions is required to better understand the cellular bases of plant development and morphogenesis.

*Arabidopsis thaliana* early embryogenesis represents an attractive model to study how the position and orientation of division planes are selected. During the first cell generations, the remarkable embryo geometry is indeed organized from a single initial cell through a stereotyped sequence of invariantly oriented cell divisions [4, 5]. Consequently, cell fate territories have been inferred and mapped through numerous genetic and cytological trace back analyses and these properties have been successfully used to identify the origin of developmental defects in patterning mutants [6, 7].

The effect of cell shape on the orientation and selection of the cleavage plane in animal and plant cells has received much attention [8] with a particular emphasis on the classical geometry-based division rules defined in the end of the 19th century [9–12]. According to Errera’s rule [12], plant cells would behave as soap bubbles so that symmetric divisions would follow a minimum interface area principle. Besson and Dumais [13] recently revisited this rule into a stochastic version according to which the selection of the cleavage plane between different alternatives obeys a probability distribution related to plane area. It is commonly accepted that the surface area minimization principle of Errera’s rule would represent a default mechanism for plant cell division in the absence of internal or external cues [14]. However, the vast majority of studies that subtend this view have focused on symmetric divisions in tissues than were assimilated to 2D systems (such as for example tissues with constant cell thickness and perfectly anticlinal divisions). Recently, Yoshida et al. [15] reported they could not identify a geometrical rule underlying the sequence of 3D cell division patterns in *Arabidopsis thaliana* embryo, when restricting division interfaces to planar surfaces. Hence, whether geometrical rules also hold for 3D plant cell division remains to be elucidated.

In the present study, we questioned the existence and nature of rules governing cell divisions in plant early embryo. Using automated image analysis of 3D embryos, we quantified cell shapes and division patterns. We found that the distance between division plane and mother cell centroid was an invariant feature throughout generations and spatial domains. We developed a computer model of cell division to investigate the space of candidate division planes under geometrical constraints in real 3D cell shapes. Using this model, we revealed a new rule for predicting the position and the orientation of division planes from mother cell geometries, which was valid for both symmetric and asymmetric cell divisions. Our results highlight a central role for a geometrical feedback loop between cell shape and division plane positioning in the self-organization of the early embryo. We report data suggesting the rule can be interpreted as surface minimization constrained by passing through the cell nucleus, and validate this interpretation using live imaging data of cell divisions in the leaf stomatal lineage.

## Results

### 3D image analysis reveals spatially organized cell shapes

**Fig 1:**
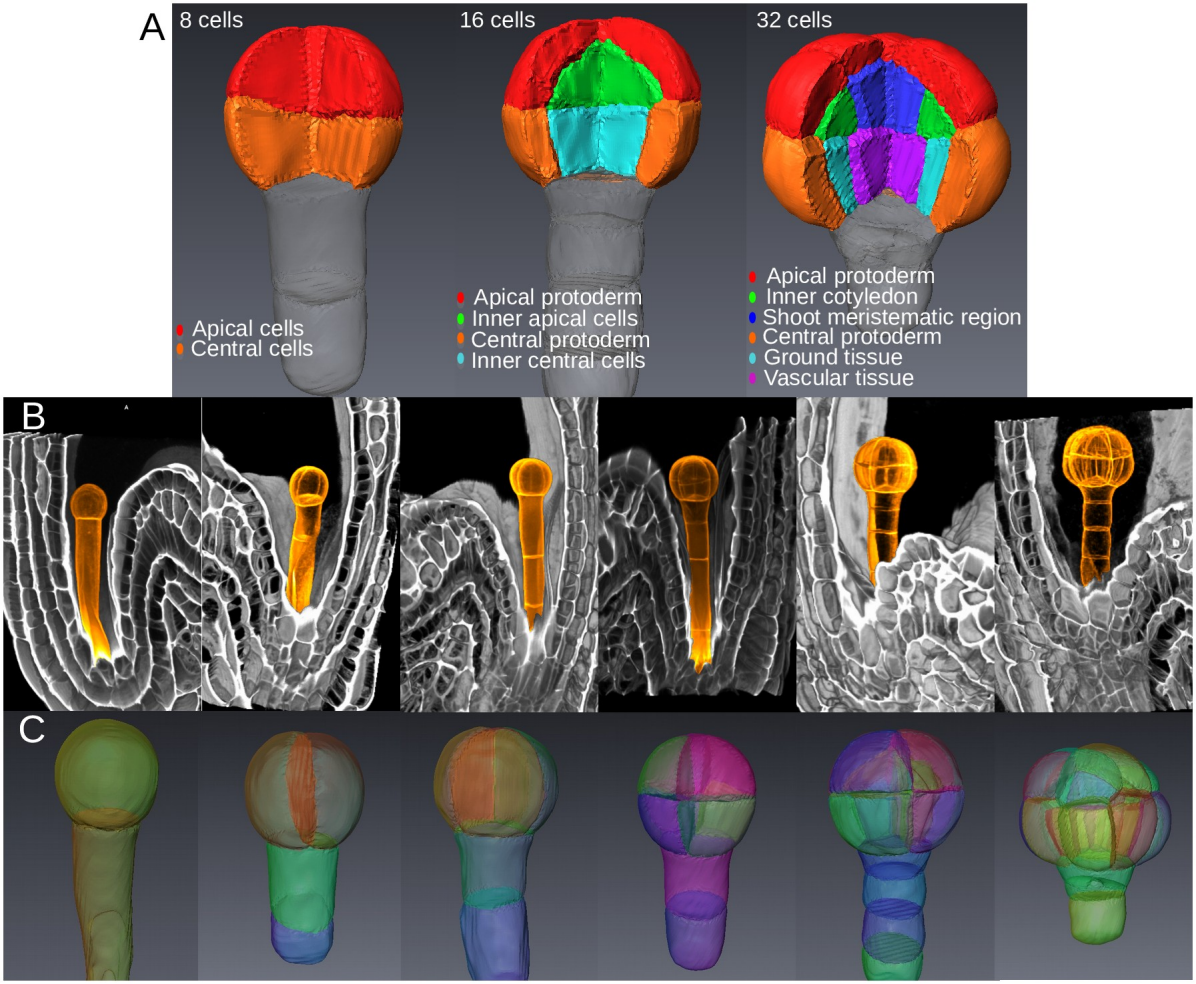
Spatial organization and description of early embryo development. (A) 3D representation of cell identity in 8C, 16C and 32C-stage embryos adapted from cytological trace-back analyses. (B) Volume rendering of 3D confocal images of embryos at all canonical stages from 1C to 32C. *Orange*: post-acquisition colorizing of embryos and suspensors. (C) Segmented cells in embryos from 1C to 32C stages.

To analyze the role of cell geometry on division plane, we used *Arabidopsis thaliana* early embryo, a model system in which almost invariant orientations of cell divisions have allowed the definition of cell fate territories (Fig 1A)[4–7]. High-resolution images of propidium-iodide-stained fixed embryos were acquired at various developmental stages up to 32C. The acquired embryos were subsequently ordered according to their number of cells (Fig 1B, S1 Fig).

**Fig 2:**
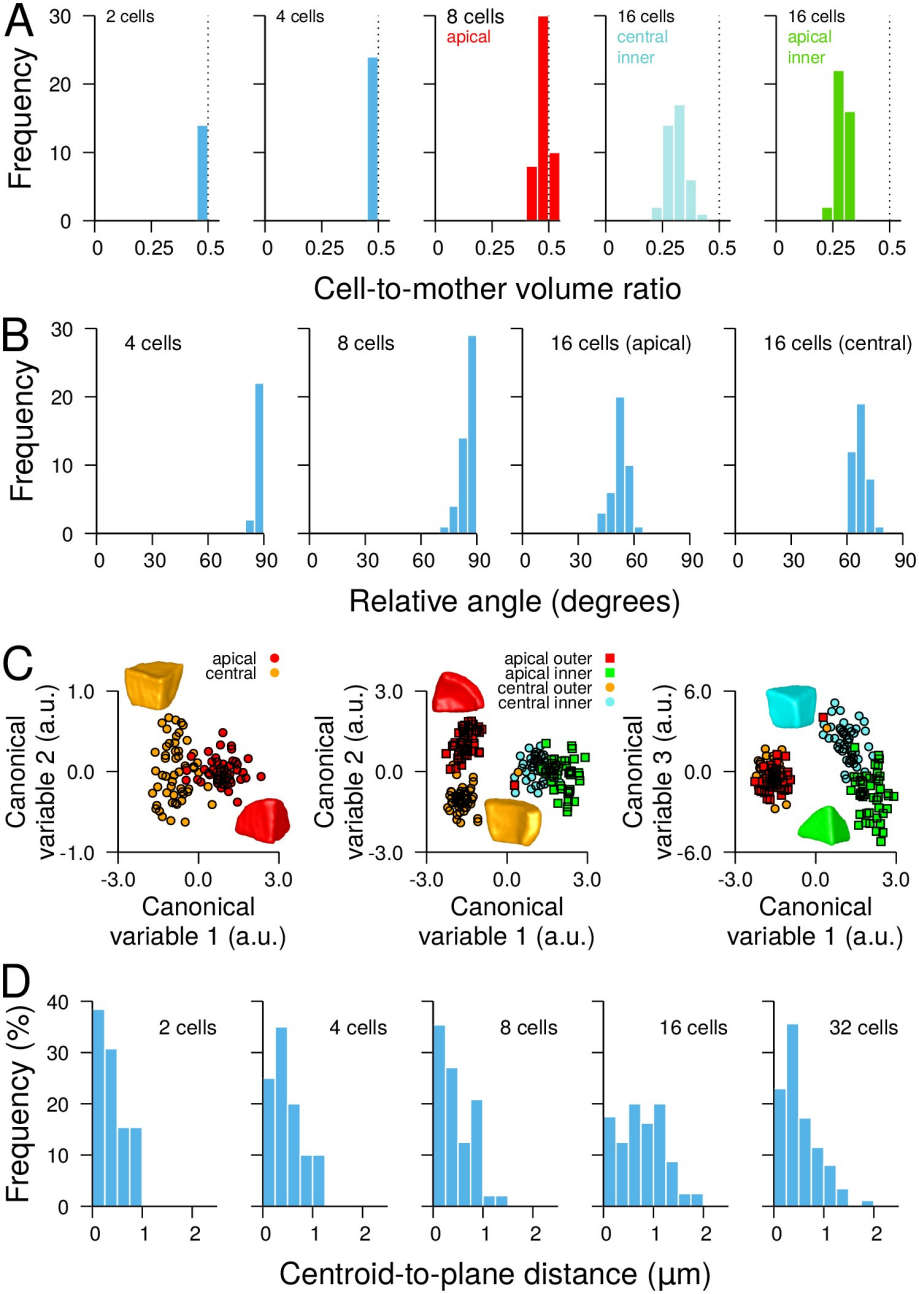
Geometric features of cell divisions at successive embryonic stages. (A) Distribution of volume ratio between daughter and mother cells. The daughter cell used in the ratio was the smallest one (2C and 4C), the apical one (8C) or the inner one (16C). The dotted line at 0.5 ratio corresponds to a symmetric division. (B) Distribution of the relative angle between consecutive division planes. Each panel gives for the indicated stage the distribution of the orientation difference between each plane and the previous one. (C) Canonical factorial cell shape analysis at 8C (*Left*) and 16C (*Middle*, *Right*) stages, discriminating apical, central, inner and outer cells (*a.u.*: arbitrary units). *Insets*: illustrative shape samples from the different embryo domains. (D) Distribution of the distance between the centroid of the mother cell and the division plane. Same color code in (A) and (C) as in Fig 1A.

The evolution over the generations of cell morphology and division plane characteristics was assessed using automated image processing and quantitative analysis. Image stacks from at least 10 embryos per canonical stage (1C, 2C, 4C, etc.) were processed and segmented to generate 3D digital reconstructions of cells within embryos (Fig 1C). Based on the chronology of invariant patterns (S2 Fig), we unambiguously inferred the cell lineage of each embryo and reassembled sister cells into mother cells. From these data, we quantitatively characterized cell divisions according to i) the morphology of mother cells, ii) the orientation of cleavage planes and iii) the volume-ratio *ρ* between sister cells and their mothers. We found that symmetric (*ρ* ≈ 0.5) (Fig 2A) and orthogonal (Fig 2B) divisions at the 2C and 4C stages gave rise to symmetric morphologies. In contrast, divisions at 8C stage were slightly asymmetric (*ρ* ≈ 0.45) with the apical daughter cell being smaller in about 80% of the cases than its apical sister (Fig 2A). The orientation of divisions at 8C was generally orthogonal to the previous division (Fig 2B). At 16C, all divisions were strongly asymmetric (*ρ* ≈ 0.3) in the central and in the apical domains (Fig 2A) and periclinal (Fig 2B). The sequence of cell division patterns was remarkably invariant up to the dermatogen stage. In both 8C and 16C stages, discriminant analysis could segregate cells from distinct embryo domains (apical vs. central; external vs. internal) using geometrical measurements (Fig 2C), showing that asymmetric divisions lead to distinct cell shapes. Overall these results show a stereotyped but complex spatio-temporal organization of cell divisions, leading to diverse cell shapes that are specific to the domains known to prefigure the future tissues of the seedling.

Between the 16C and 32C stages, asymmetric periclinal divisions in the inner central domain defined an additional cell layer prefiguring the vascular tissue [7] while anticlinal symmetric divisions occurred in protoderm (S1S–S1U Fig). In the central domain, each mother cell divided invariantly in terms of shape, volume ratio and division plane orientation, thus preserving the radial symmetry established in the previous generations (S1T Fig; S3A–S3B Fig). In the apical domain, outer cells displayed variable orientation of anticlinal division (S1U Fig; S3C Fig). Inner apical cells displayed a bimodal distribution corresponding to two asymmetric volume ratios giving rise to four different cell shapes (S4 Fig; S3D and S3E Fig). This variability in cell division pattern led to a loss of radial symmetry in the apical domain. Volume-ratio was also very variable at the embryo scale, but exhibited specific distributions within each cell category (S4 Fig). This further confirmed that each spatially distributed cell type shares specific volume-ratio characteristics, specific cell geometry and division plane orientation.

### The division plane passes through the mother cell centroid

Because cell division patterns lead to cell populations with domain-specific geometries, we wondered whether intrinsic properties of cell division, other than volume-ratio and plane orientation, would be invariant irrespective of cell shape. We evaluated whether the division plane passed through the centroid of the mother cell, one of the features derived from Hertwig’s rule on symmetric cell division in animal cells [8, 11]. From the segmented images, we extracted the surfaces of the division planes between sister cells and we computed the centroids of the reassembled mother cells. The distance between mother cell centroid and division plane was remarkably invariant across generations and domains in the embryo. The distribution of this distance from 2C to 32C stages was indeed concentrated in the 0-1 *μ*m interval (Fig 2D), with average values (2C: 0.40±0.08 *μ*m, *n* = 14; 4C: 0.62±0.15 *μ*m, *n* = 24; 8C: 0.47±0.05 *μ*m, *n* = 48; 16C: 0.78±0.05 *μ*m, *n* = 80; 32C: 0.52±0.04 *μ*m, *n* = 87) close to the imaging resolution (voxel diagonal: 0.61 *μ*m). This suggested that in symmetric as well as in asymmetric divisions the cleavage plane was passing through, or at least very close to, mother cell centroids, which was further confirmed by visual examination of 3D reconstructions (S5 Fig). This invariance led us to hypothesize that despite the large differences in cell geometry, division volume-ratio, and plane orientation, a common law could underlie the selection of cleavage planes during the early generations of *Arabidopsis thaliana* embryogenesis.

### A new computational 3D model reproduces the geometrical features of observed division planes

Given the observed invariance of the plane distance to mother cell centroid, we hypothesized that common constraints were exerted on the selection of the division plane. We questioned whether the minimum area principle of Errera’s rule [12], which has been shown to explain a number of division patterns in plants tissues [13], was also satisfied in early embryo 3D cells. We designed a computational model in which cell division plane was obtained by the stochastic minimization of an energy function *H*(*x*) over the set {*x*} of all possible binary partitions of the mother cell, using a discrete representation of the 3D space (S6A Fig). To simulate divisions that obey area minimization at arbitrary volume-ratios, the energy was defined so as to simultaneously satisfy a volume-ratio constraint and an interface area constraint:

$$\begin{array}{c} H\left(x\right)=H_{V}\left(x\right)+H_{A}\left(x\right) \end{array}$$

The only free scalar parameter of the model was ρ*, the target volume-ratio of the simulated divisions. Configurations with a volume-ratio distinct from ρ* were penalized by defining *H*_*V*_(*x*) as an increasing function of the square difference between ρ* and the actual volume ratio *ρ*(*x*) of partition *x*. Configurations with large interface areas were penalized by defining *H*_*A*_(*x*) as an increasing function of the interface area in partition *x*. Starting from arbitrary random partitions *x*, the minimization of *H* thus yielded solutions satisfying as much as possible the surface minimization and volume ratio constraints (S6B Fig). The model was first validated by running symmetric divisions (ρ* = 0.5) within simple synthetic shapes. Partitions of spheres, half-spheres, and quarters of spheres all matched theoretical expectations (S7 Fig).

**Fig 3:**
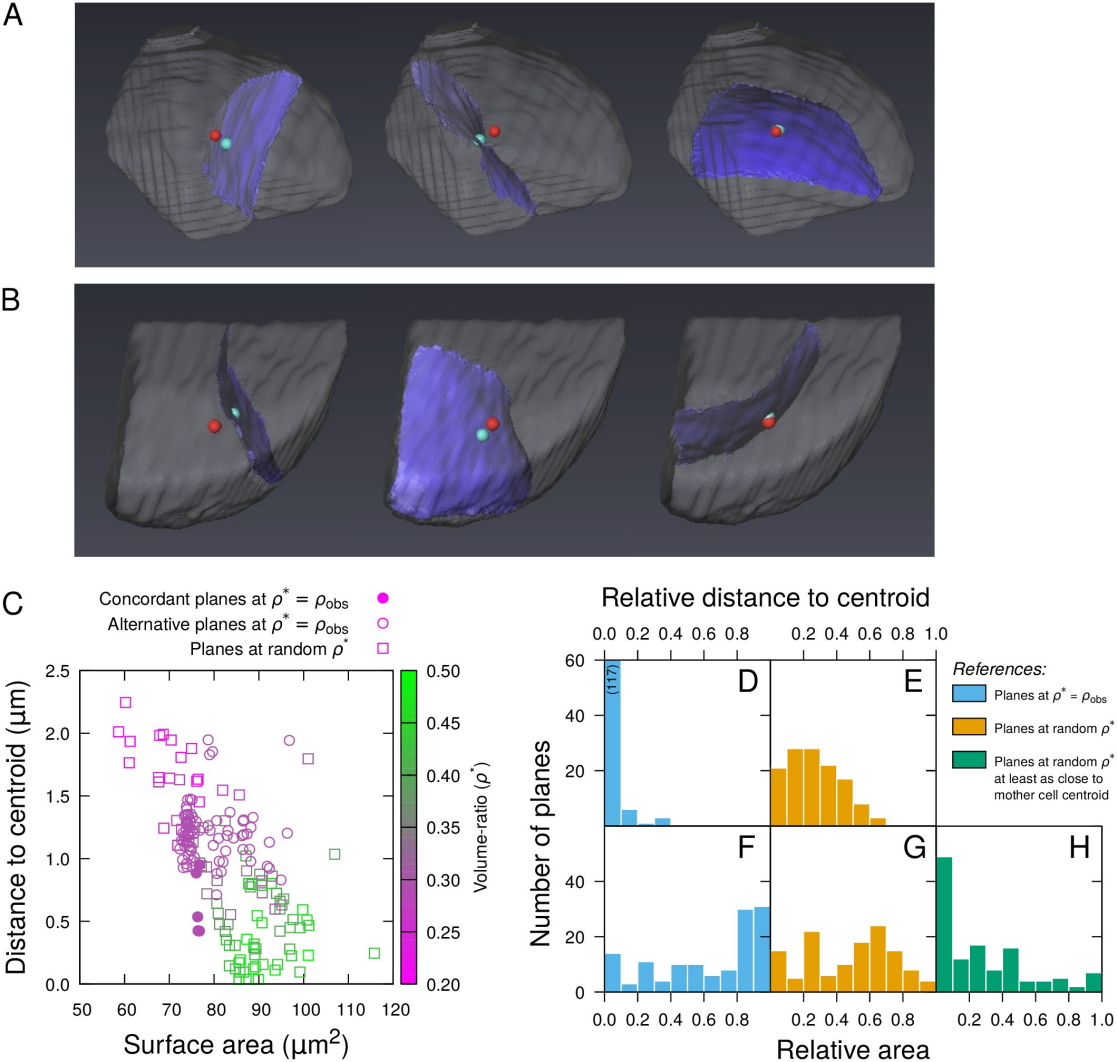
Modeling asymmetrical divisions at the 8C-16C transition. (AB) Results of three independent simulations of asymmetric divisions in real apical (A) or central (B) cells reconstructed at the 8C stage. The red dot indicates the mother cell centroid, the blue dot corresponds to the closest point on the division plane. (C) Distance to centroid as a function of surface of area of simulated division planes in a sample mother cell. (D-H) Relative distance to mother cell centroid (DE) and relative area (F-H) of simulated planes reproducing observed patterns in 10 mother cells. The relative measurements (detailed in Material and methods) were obtained by comparison with reference datasets composed of either all alternative planes obtained at observed volume-ratios (*Blue*), all planes obtained at random volume-ratios (*Orange*), or planes passing as close to cell centroids among those obtained at random volume-ratios (*Green*).

We applied the model to simulate asymmetric cell divisions at the dermatogen stage (8C-16C transition), where the most asymmetrical volume-ratios were observed (Fig 2A). The model was run on real apical (*n* = 11) and central (*n* = 9) mother cells reconstructed at the 8C stage by merging sister cells from as many distinct 16C segmented embryos. In each case, the target volume ratio ρ* was set to the real volume-ratio *ρ*_*obs*_ measured on the sister cells prior to merging. Because of its stochastic nature, the model was run several times on each mother cell to explore the solution space induced by *H*. Out of 40 simulations for each cell, the model produced several types of divisions: some were never observed in real embryos while others (subsequently referred to as *concordant* simulated planes) reproduced curvature, right-angle intersection with pre-existing cellular walls, and periclinal orientation of the division patterns observed at this stage (Fig 3A and 3B).

To evaluate the validity of the area minimization principle, we compared the area of concordant planes to the alternative solutions obtained in the same cell. This comparison suggested that the concordant planes did not exhibit smaller areas compared with alternative ones (Fig 3C). Raw measurements from different cells could not be pooled because of cell size differences (a relatively small area in a large cell can be larger than a relatively large area in a small cell). We thus applied a normalization procedure by computing for each concordant plane the proportion of alternative simulated planes from the same cell that had a smaller area (see Materials and methods). This relative area varied between 0 (plane smaller than every other plane it is compared to) to 1 (plane larger than every other one). The distribution of their relative areas revealed that concordant planes had on average higher surface areas than all other plane types (Fig 3F). We concluded that the minimal area principle alone could not account for the asymmetric periclinal cell division patterns observed at this stage.

### Division planes can be predicted by distance-to-centroid and surface area constraints

We further examined the distance to mother cell centroid for the periclinal simulated division planes. We found it was on average close to image voxel size (apical: 0.31±0.03*μ*m; central: 0.49±0.07 *μ*m; Fig 3C). Given the limit imposed by the spatial discretization on the distance to the centroid, we concluded that the model accurately reproduced periclinal divisions passing through the centroids of mother cells. Using per-cell normalization as above for area, we observed that the centroid-to-plane distance was relatively lower for periclinal planes compared with all other types of simulated planes (Fig 3D). For these other types of planes, the average distance to centroid (apical: 1.17±0.02 *μ*m; central: 1.16±0.02 *μ*m) showed they were not passing through the centroid, as confirmed by 3D renderings (Fig 3A and 3B).

We next compared periclinal division planes to other types of division planes also passing through cell centroids. To this end, we simulated symmetric divisions by setting ρ* = 0.5. We obtained several solutions with centroid-to-plane distance close to the voxel size (apical and central, average distance: 0.32±0.01 *μ*m), thus confirming that symmetric divisions shared with asymmetric periclinal divisions the characteristic of passing through the mother cell centroid. However, no periclinal solution was obtained in the symmetric case. We then examined the validity of the surface area minimization principle between asymmetric periclinal divisions and symmetric ones. Remarkably, the area of asymmetric periclinal planes was comparatively lower than the area of symmetric division planes (S8 Fig). Altogether, our modeling results suggested that observed periclinal divisions corresponded to division planes that pass through the centroid of the mother cell and, given this constraint is satisfied, minimize the interface area between daughter cells.

To further evaluate the validity of this hypothesis, we checked that periclinal solutions at observed volume ratios were globally optimal according to the new rule by comparing them to alternative solutions obtained over a large range of alternative volume ratios. To this end, we ran the model 100 times in each mother cell; for each run, ρ* was drawed from a uniform distribution between 0.2 and 0.5, a range that covered observed volume-ratios in asymmetric and symmetric divisions (Fig 2A). We ran an additional set of 100 runs with ρ* set to the measured value *ρ*_*obs*_ for each mother cell and compared the periclinal divisions obtained in this set to the alternative solutions at random ρ*. We found that periclinal solutions at ρ* = *ρ*_*obs*_ did not globally minimize area (Fig 3G) but they minimized the distance to the centroid (Fig 3E). Restricting the comparison with the solutions obtained at random *ρ* that had a smaller or equal distance to the centroid revealed that the concordant planes also minimized area, conditioned on the minimization of the distance to the centroid (Fig 3H). This analysis generalized the results obtained when comparing simulations between ρ* = *ρ*_*obs*_ and ρ* = 0.5. It showed that a nested rule, imposing the passing through or close to the centroid and then the minimization of surface area, predicted the positioning of the division planes at the 8C-16C transition.

### The new geometrical rule is valid at each generation

**Fig 4:**
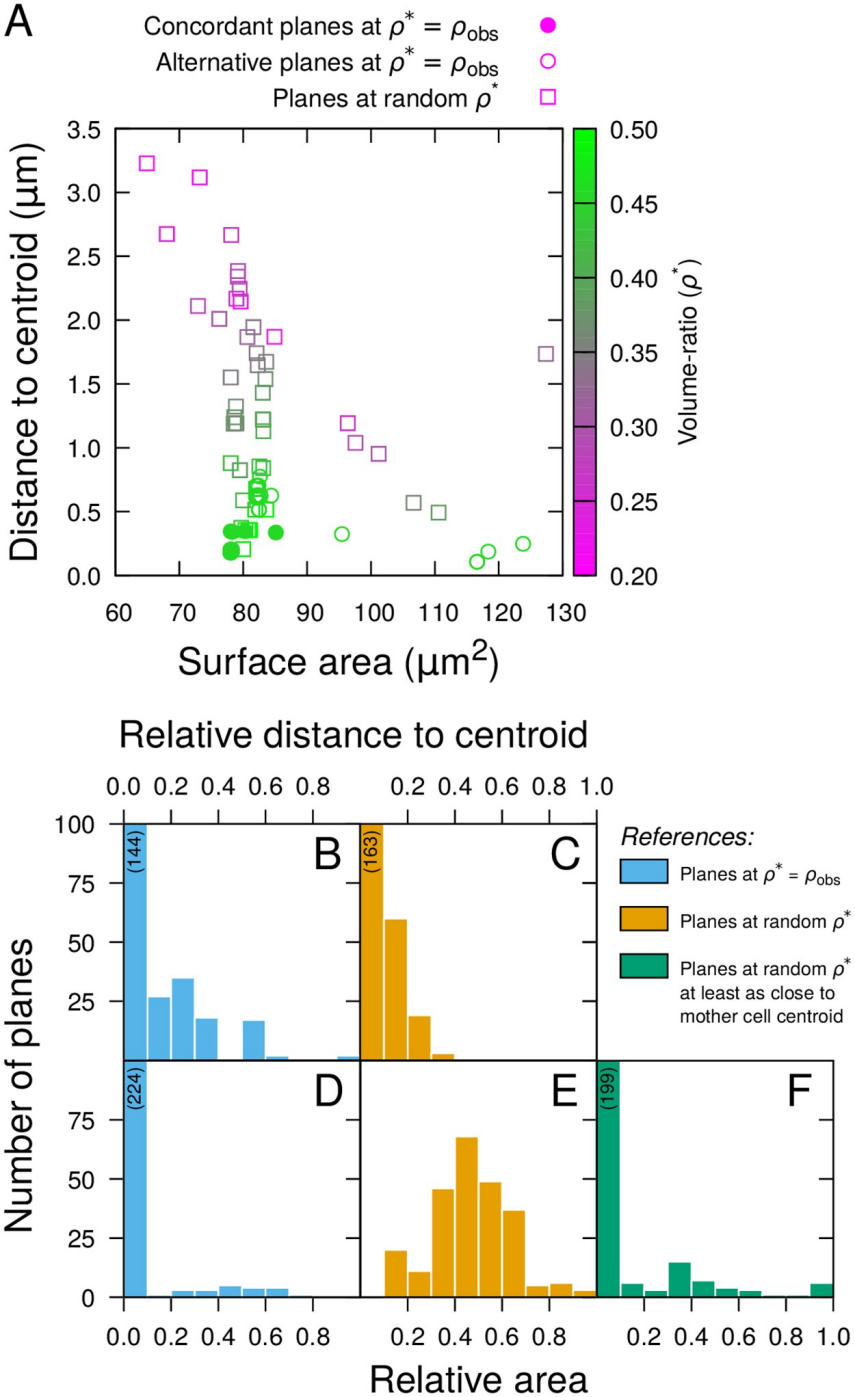
Evaluation of the geometrical rule at the 4C-8C transition. (A) Distance to centroid as a function of surface of area of simulated division planes in a sample reconstructed mother cell from the 4C stage. (B-F) Relative distance to mother cell centroid (BC) and relative area (D-F) of simulated planes reproducing observed patterns in 10 mother cells. The relative measurements were obtained by comparison with reference datasets composed of either all alternative planes obtained at observed volume-ratios (*Blue*), all planes obtained at random volume-ratios (*Orange*), or planes passing as close to cell centroids among those obtained at random volume-ratios (*Green*). Numbers in parentheses indicate heights of truncated histogram bars.

We applied the model at the transition from 4C to 8C generations, where volume-ratios were slightly asymmetrical, the central cell being generally larger than the apical cell (Fig 2A). We reconstructed 10 mother cells by merging sister cells from as many distinct 8-cell embryos. We ran the model 50 times in each cell, setting ρ* to the corresponding measured volume ratio *ρ*_*obs*_. The vast majority of simulated planes were distributed between two main types, both oriented transversally but differing in the location (central or apical) of the largest cell (S9 Fig). Longitudinal planes were obtained only occasionally. The planes reproducing observed patterns (concordant planes: transverse division with largest cell in central location) presented a relatively lower distance to the mother cell centroid compared with all alternative solutions, as evidenced in per cell analysis (Fig 4A) as well as in population analysis (Fig 4B). The distance to centroid for the concordant planes was again of the order of voxel size (average 0.24±0.01 *μ*m; Fig 4A), demonstrating that these planes were passing through, or close to, the centroid (S9 Fig). The concordant solutions also presented a smaller area than the alternative planes (Fig 4A and 4D). This contrasted with the results obtained at the 8C-16C transition and was probably due to the fact that asymmetry was less pronounced at the 4C-8C transition.

To evaluate whether the concordant solutions at the 4C-8C transition corresponded to global optima of the two-step geometrical rule over a large range of volume-ratios, we ran 50 additional simulations in each reconstructed mother cell with ρ* drawn uniformly at random between 0.2 and 0.5. The relative area of concordant division planes obtained at ρ* = *ρ*_*obs*_ compared with the area of planes obtained with random ρ* had a large dispersion around 0.5 (Fig 4E), showing that, as for the 8C-16C transition, area alone was not sufficient to predict the observed division patterns. By contrast, the distance to the centroid of the concordant simulated planes was relatively smaller compared with most of these alternatives (Fig 4C). Some planes obtained at random ρ* were also passing close to the mother cell centroid (Fig 4A). When compared to these, the concordant simulated planes had a relatively lower area (Fig 4F). The few simulated planes obtained at random ρ* that had similar area and distance to centroid compared with concordant planes obtained at ρ* = *ρ*_*obs*_ (Fig 4A) were actually also reproducing the patterns observed in the embryos, and corresponded to sampled values of *ρ* close to *ρ*_*obs*_. Altogether these results show that, at the 4C to 8C transition, the two-step rule (minimize distance to centroid, then interface area) faithfully predicted the position of the division plane, while the area minimization principle alone did not.

Running the model on the 1C to 2C and on the 2C to the 4C rounds of divisions with ρ* set to observed volume-ratios yielded several solutions. At both generations, we observed no difference in the distance to mother cell centroid between simulated planes reproducing the orientation of actual division planes and alternative ones (S10B Fig and S11B Fig), but concordant planes presented a smaller plane area (S10D Fig and S11D Fig). However, their area was on average larger, and their distance to centroid was much smaller, from that of solutions obtained by running the model with volume-ratio randomly set between 0.2 and 0.5 (S10C and S10E Fig and S11C and S11E Fig). Compared with solutions under random ρ* passing as close or closer to mother cell centroid, the concordant simulated planes had a lower area (S10F Fig and S11F Fig). Hence, concordant solutions again uniquely corresponded to the minimization of the distance to the centroid and of the interface area. Overall, the same rule predicted the selection of observed division planes between consecutive generations from the initial apical cell up to the 16 cells stage (S12 Fig).

**Fig 5:**
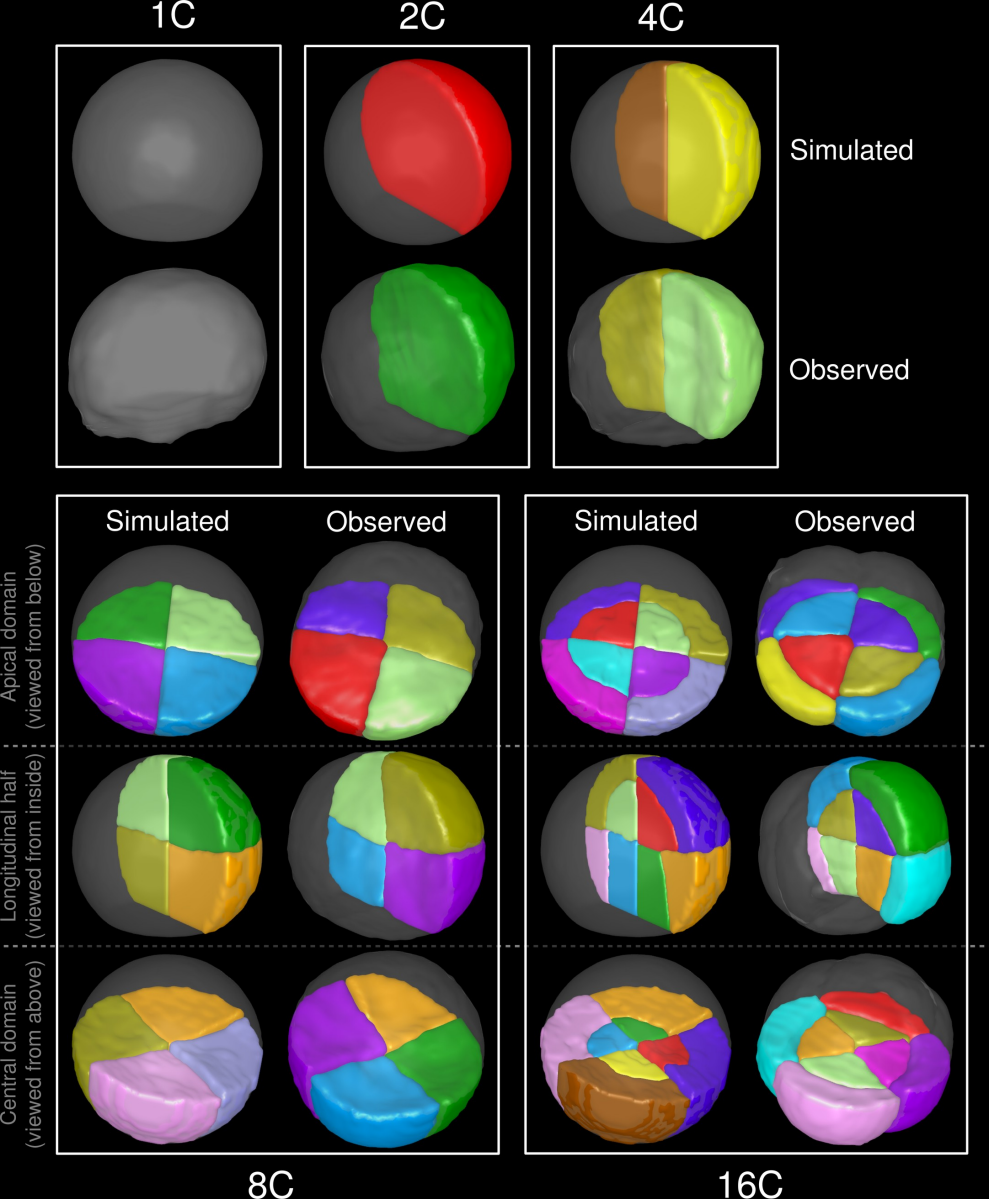
The geometrical rule reproduces the complete sequence of events up to the 16C stage. Results of recursively applying the rule from an initial truncated sphere (*1C, Top*). The model was run 50 times at each generation, with volume-ratio ρ* set to 0.5 (1C-2C and 2C-4C transitions), 0.45 (4C-8C) and 0.33 (8C-16C). The best simulation according to the rule was selected as input for the next generation. Cellular patterns observed on real embryos from the the corresponding stages are shown for comparison.

We evaluated the capacity of the proposed rule to reproduce observed division patterns over several successive generations. We ran 50 simulations of the model within a truncated sphere representing the 1C embryonic stage and selected, among the solutions passing through or close to the centroid (distance below 1 voxel), the solution of least surface area. We then repeated recursively the process until the 16C stage. The obtained patterns faithfully reproduced the stereotypical global cellular organizations at all stages (Fig 5), demonstrating the ability of the rule to predict, from the initial apical cell alone, the sequence of cell division events during the first generations of *A. thaliana* embryogenesis. Some differences in geometry could be observed at the final stage in the central domain, where the orientation of the simulated interfaces, parallel to the first division plane, slightly differed from the observed ones, which were generally parallel to the outer cellular walls (Fig 5, 16C). These fine-scale differences were likely due to the fact that cellular growth during the considered generations was not considered in our model.

### The positions of nuclei coincide with mother cell centroids

We questioned the biological basis of the constraint on the centroid-to-plane relative position. In most plant cells, the future position of the division plane can be inferred prior to division from the positioning of the pre-prophase band, a cortical ring of microtubules and actin at the cell periphery around the nucleus [1, 16]. Consequently, the position of the nucleus could guide the future division plane in both symmetric and asymmetric division. We combined nucleus and cell wall staining [17] and generated segmented embryos from which the centroids of cells and of nuclei were determined. We observed a close correspondence between cell centroids and nuclei (S13A Fig). The centroid-to-centroid distance between nuclei and cells was concentrated in the range 0-1 *μ*m with an average of 0.68±0.04 *μ*m (S13B Fig), which was similar to the measured distances between cell centroids and division planes (Fig 2D). We concluded that nucleus positioning may obey geometrical constraints and could provide the intermediate link between cell geometry and division plane selection.

Based on these results, we reasoned that we should be able to predict division planes using area minimization conditioned on passing by or close to the nucleus centroid. We evaluated this possibility by running the model on 8C stage embryos with stained nuclei and cell walls. We ran the model 500 times in each cell and selected the solutions corresponding to periclinal planes passing at most at one voxel distance from the nucleus centroid (S13C Fig). Compared to all alternative solutions, these periclinal solutions had a relatively smaller distance to the nucleus centroid (S13D Fig). They also had a smaller interface area compared with alternatives passing at least as close to the nucleus (S13E Fig). Because of the concordance between periclinal solutions and the stereotyped patterns observed in real embryos at this stage, these results suggest that the positioning of division planes can indeed be predicted based on the position of the nucleus and area minimization.

### Area minimization constrained on nucleus positioning predicts division patterns in epidermal leaf cells

**Fig 6:**
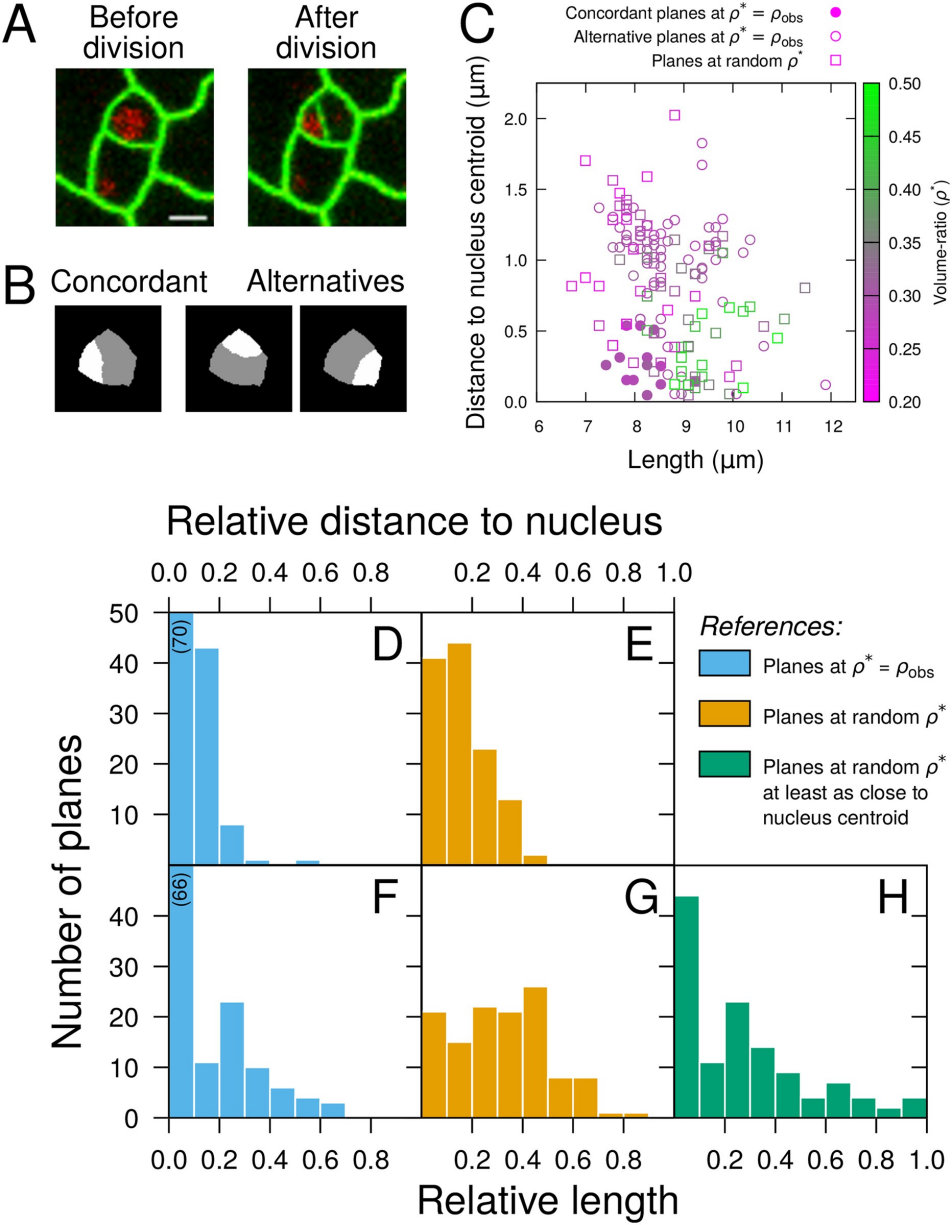
Evaluation of the geometrical rule in the stomatal cell lineage. (A) Time-lapse imaging of leaf epidermal cells (*Green*: LTI6B-GFP; *Red*: H2B-RFP). Scale bar: 5 *μ*m. (B) Sample simulated divisions of the dividing cell shown in (A), reproducing (*Concordant*) or not (*Alternatives*) the actual division. (C) Distance to nucleus centroid as a function of length for division planes simulated in this same cell. (D-H) Relative distance to nucleus centroid (DE) and relative length (F-H) of simulated planes reproducing observed divisions in 7 mother cells from the stomatal lineage. The relative measurements were obtained by comparison with reference datasets composed of either all alternative planes obtained at observed volume-ratios (*Blue*), all planes obtained at random volume-ratios (*Orange*), or planes passing as close to nucleus centroids among those obtained at random volume-ratios (*Green*). Numbers in parentheses indicate heights of truncated histogram bars.

Our results in early embryos suggested the rule could be reformulated as a minimization of division plane constrained on the passing through the cell nucleus. To further test this hypothesis, we evaluated whether division planes could be predicted based on cell geometries and nucleus positions in leaf epidermal cells from the stomatal cell lineage, which present asymmetric divisions associated with nucleus migration away from a central position [18]. Using time-lapse recordings (Fig 6A), we analyzed nucleus positions immediately prior to cell divisions and measured volume ratios in daughter cells immediately after. Due to the low contrast at bottom cell boundaries in the recorded movies, image segmentation and measurements were performed in 2D. Since in the leaf epidermis division planes are anticlinal and run parallel to each other orthogonally to the surface, this was sufficient to capture the geometry of cells and divisions. The observed volume-ratio *ρ*_*obs*_ covered a large range corresponding to asymmetric divisions (0.28 ≤*ρ*_*obs*_≤ 0.45; average 0.35±0.02, n = 7) and the nucleus was generally not centrally located (average distance between cell and nucleus centroids: 1.51±0.28 *μ*m, n = 7).

A 2D version of the computational division model was run within each mother cell mask. As in the 3D embryo cells, running the model with ρ* set to *ρ*_*obs*_ produced solutions from a limited repertoire of possibilities, one of which reproduced the positioning of the observed divisions (Fig 6B). These concordant solutions were quantitatively compared to the alternative ones and to simulated divisions obtained when running the model at random ρ*. The distance between the division interface and the nucleus centroid was comparatively smaller in concordant solutions (Fig 6C–6E), whereas the division interface length did not show a comparable trend when compared to solutions at random ρ* (Fig 6C, 6F and 6G). However, the concordant solutions minimized this length when compared to the solutions obtained at random ρ* that passed at least as close to the nucleus (Fig 6H). These observations show that, in leaf epidermal cells, divisions are consistent with the rule of plane area minimization constrained on the passing through the cell nucleus.

## Discussion

The first generations of *A. thaliana* embryogenesis are characterized by stereotyped cell division patterns that present different orientations and volume-ratios between successive generations. Combining quantitative image analysis and a new 3D computational model of cell division, we report here an invariant positioning of division planes relative to mother cell geometries and show that a single geometrical rule linking cell geometry to division plane characteristics is able to explain the complete sequence of observed stereotyped patterns. Our data further suggested the rule could be interpreted based on area minimization constrained by the position of the nucleus, which was validated in leaf epidermal cell divisions.

In the embryo, where the nucleus occupied a central position, the rule predicts the division plane as the minimum area surface among those passing through the mother cell centroid. The rule manifests itself statistically by skewed distributions of the relative distance to the cell centroid and of the relative surface area when comparing simulated planes reproducing observed patterns to alternative solutions. The candidate sources of noise in these distributions include experimental procedures, residual segmentation errors, and differential cell growth. We strove to minimize the first two by discarding samples altered during experimental preparation and by visually checking all segmentations. An open remaining question is the potential impact of differential growth between cell division and image acquisition. When they become compatible with cell-resolved 3D image segmentation, live-imaging protocols allowing to dynamically track cell divisions in the early embryo [19] will be essential to address this issue. In our analysis of leaf epidermal cell divisions, the noise level was comparable to that observed in the embryo study. The reduced uncertainty due to cell growth that was gained thanks to live imaging was likely compensated by the necessity to process in 2D images with a lower resolution compared with embryo data.

Most of previous modeling studies on plant division rules have focused on the symmetric case and in tissues where cell geometries can be assimilated or reduced to essentially 2D shapes such as the thallus of green algae or the epidermal layer of the shoot apical meristem [13, 20–24]. Geometrical approximations (modeling divisions as rectilinear segments of minimal lengths passing through the cell center) of the rule proposed here have been considered in 2D simulation studies. Sahlin and Jönsson [22] showed this rule was able to statistically reproduce at the tissue scale some of the topological and geometrical features of cellular organizations. In a theoretical study, Alim et al. [25] showed the same rule could regulate growth heterogeneity at the tissue scale. Our study complements these works and brings new insights in the study of plant cell division principles by demonstrating the validity of this rule in a three dimensional system and in individual cells. As methods for the 3D/3D+t reconstruction of plant tissues are increasingly available [26–28], one perspective of the present study is to evaluate the ability of our model to reproduce statistical properties of 3D cellular organizations in tissues such as the shoot apical meristem.

The geometrical rule demonstrated here to be predictive of cell division patterns in the early embryo and the central positioning of the nucleus can be interpreted based on the known dynamics of the cytoskeleton. Before cell division, cytoplasmic strands radiating between the nucleus and the cell periphery appear and dynamically coalesce to form the phragmosome [29]. These strands, containing actin [30] and microtubules [31], are under tension [32, 33]. Because the phragmosome prefigures the position of the future division plane, Lloyd [34] proposed this tension could provide the mechanical basis for plane area minimization, an interpretation that was adopted by Besson and Dumais [13] in their stochastic formulation of Errera’s rule. The same mechanism can more generally explain our geometrical rule because it predicts both the central positioning of the nucleus and the minimization of the division plane area. Because symmetric divisions with minimum area also passed through the mother cell centroid, Errera’s rule appears as a particular case of the more general two-step rule revealed here, in which the relative position of the division plane to the mother cell centroid takes precedence over area minimization. This is consistent with a common mechanism providing an interpretation for these two rules.

Symmetric division according to the area minimization principle is often viewed as a default regime, whereas asymmetric division is considered to require specific cues such as hormonal signals [1, 14, 15, 35]. Introducing a model accounting for symmetric as well as for asymmetric cell divisions in actual 3D cell shapes, our study in the early embryo deviates from this paradigm by showing that a single geometrical rule can explain a large spectrum of division patterns, from highly symmetric (first two generations) to slightly (third generation) or strongly (fourth generation) asymmetric divisions. Starting from the original apical cell, recursively applying the geometrical rule predicted the sequence of division patterns observed during the first generations. The resulting cell architecture of the *Arabidopsis* embryo at dermatogen stage could thus be interpreted as a self-organized structure emerging from a geometrical feedback loop between cell geometry and division plane positioning: through the geometrical rule, a given cell geometry would determine a given division plane, which in turn determines the geometry of the daughter cells, etc. The parsimony of this process contributes to its robustness, as division patterns are spontaneously directed towards a single pattern of cell organization. No specific signals were invoked to reproduce the sequence. A corollary is that the distinct fates of cells issued from asymmetrical divisions at stages 8C and 16C could result from extrinsic rather than intrinsic cues [3], raising the intriguing possibility that some of the well-established gene patterns at these stages could be determined by cellular organization rather than orchestrating it.

Mechanical cues have been shown to influence the orientation of division planes in plant tissues [36]. Louveaux et al. [24] recently proposed that symmetric division planes in the shoot apical meristem would be positioned according to tensile stress. In isotropically growing tissues, geometrical rules would hold because of cell shape-derived tension. At organ boundaries, tension would instead derive from tissue anisotropic growth and geometry, thus overriding the influence of cell shape. Our study indicates that cell geometry alone can explain the division patterns observed during early development. It will be interesting in the future to examine how this can be related to the patterns of mechanical constraints at the single and at the multicellular scales in this particular context.

Our results on intra-cellular nucleus positioning in embryo cells are consistent with an interpretation of the rule whereby the cell centroid would be a proxy for the nucleus position. The rule could thus be reformulated as area minimization conditioned by nucleus position. Several authors described a migration of the nucleus towards a central location prior to a symmetrical division [1, 29, 37]. However, in other systems such as the first division of the zygote [38], lateral root initiation [39] and stomatal complex formation [18, 40], nucleus displacement creates a polar asymmetry that would drive asymmetric divisions [41]. In meristemoids, Robinson et al. [42] showed elegantly how cell geometry and polarity cues could account qualitatively for cell lineage patterning by modeling a 2D shortest path rule through the displaced nucleus. There, however, the minimization of division plane area was assumed a priori and the position of the nucleus in the model was optimized independently of observed positions. The results reported here with the 2D version of our division model complement that study by demonstrating the minimization of the division plane constrained by nucleus positioning. We believe our modeling framework should be useful to decipher the integration into morphogenetic processes of mechanisms and cues such as growth, polarity and mechanical constraints.

Following the logic of the proposed rule, both the distance to the cell or nucleus centroid and the area of the division plane would be determined by the cellular machinery and the volume-ratio would result from these constraints. The present version of our model does not implement this logic, since the volume-ratio for a given simulation (input parameter ρ*) is imposed while the distance to the centroid is not constrained. A practical implication for the present study was that the rule was applied *a posteriori*, by checking that it allowed to select the concordant simulated planes within the space of possible solutions over all volume-ratios. This procedure ensures that the demonstrated rule is not subservient to the volume-ratio imposed in any model simulation—actually, it allows to predict the volume-ratio. A key advantage of setting the volume-ratio in simulations is to avoid degeneracy to implausible solutions (one daughter cell with null volume). Volume-ratio constraints are inherent to all historical division rules [9, 10, 12] and to their modern counterparts in mathematical or computational models [13, 21, 22]. For example, the area minimization principle embedded in Errera’s rule would also lead to degenerate patterns without conditioning on a symmetrical division; the same holds for Sachs’ and Hofmeister’s rules. One challenge for future research will be to identify the biological constraints to incorporate into modeling to prevent degeneracy and to release the volume-ratio priors in cell division models, including ours.

Lattice-based discrete space representations have been intensively used to model supra-cellular collective behaviors in animal tissues, as with the popular Cellular Potts Model [43, 44], but have been seldom adopted in studies of plant development and morphogenesis [20, 45], which preferentially relied on polygonal and polyhedral representations of cells (for review, see [46]). The voxel-based formalism introduced here for modeling plant cell division was key to reveal a geometrical rule behind observed patterns. Based on the modeling of 3D division interfaces as planar surfaces, a recent study indeed concluded that asymmetric divisions in *Arabidopsis thaliana* early embryos at dermatogen stage could not be predicted from cell geometry [15]. The discrepancy with our conclusions likely results from the larger space of candidate solutions that could be explored with our model. By removing the restriction to planar surfaces, the model could reproduce curvature and right angle intersections with pre-existing cellular walls, and consequently positioning, of observed division planes. Discrepancies between model predictions and experimental observations that are attributable to simplified cell geometries as polygonal shapes have also been reported in other systems [42]. Thus, we believe our modeling framework should be useful when the resolution of geometrical information provided by experimentally acquired images should be preserved in the models.

The new geometrical rule failed to capture some fine-scale features, such as the exact angles between cellular walls when simulating the dermatogen stage from an initial single cell embryo modeled as a truncated sphere. This suggests that, while cell division would be sufficient to explain the global architecture of the embryo at these stages, cell growth could contribute to the determination of its precise geometry. The discrete-space model, which can be extended to combine cell division and cell growth mechanisms, will be useful to explore this hypothesis as well as to decipher the coordination between these two mechanisms in the evolution of embryo shape beyond the dermatogen stage. In addition, the stochastic nature of the model will make it a useful tool to interpret the phenotypic variability of division patterns and its impact on tissue organizations beyond the 16C stage and in contexts where early embryogenesis is not as stereotyped as in wildtype *Arabidopsis* embryos.

### Addendum

While the revised version of the present paper was under review, Martinez et al. [47] published a 3D model of symmetric cell divisions and Chakrabortty et al. [48] reported how they attempted to predict the global orientation of division planes in the embryo based on the organization of cortical microtubules.

## Materials and methods

### Plant material and growth conditions

*Arabidopsis thaliana* ecotype Col0 was used as the wild type. Plants were grown in greenhouse with 14 hours of light and a temperature of 19° C by day and 16 °C by night. Siliques were harvested one by one from the first one (which had a longer pistil than the unfertilized flowers) to the fifteenth.

Transgenic plants containing P35S:H2B-RFP/P35S:GFP-LTI6B [49] were used for live imaging acquisition of leaf epidermal cells. Seeds were sown on Arabidopsis medium (Duchefa) as described previously [50] supplemented with 1% sucrose. After 48h at 4° C in darkness, plants were grown under long day conditions in Petri dishes (16h light/8h dark cycle; 60% humidity; 19 °C day and night).

### Sample preparation and imaging

#### mPS-PI staining

Siliques were opened and fixed in 50% methanol and 10% acetic acid five days at 4° C. Samples were rehydrated (ethanol 50%, 30% 10% and water) then transferred 3 hours in a 0.1 N NaOH 1% SDS solution at room temperature. Next, samples were incubated 1 hour in 0.2 mg/ml *α*-amylase (Sigma A4551) at 37° C [51]. After, samples were bleached in 1.25% active Cl^−^ 30 to 60 seconds. Samples were incubated in 1% periodic acid at room temperature for 30 min and colored by Schiff reagent with propidium iodide (100 mM sodium metabisulphite and 0.15 N HCl; propidium iodide to a final concentration of 100 mg/mL was freshly added) overnight and cleared in a chloral hydrate solution (4 g chloral hydrate, 1 mL glycerol, and 2 mL water) few hours. Finally, samples were mounted between slide and cover slip in Hoyer’s solution (30 g gum arabic, 200 g chloral hydrate, 20 g glycerol, and 50 mL water) using spacers.

#### Cell wall and nucleus staining

Siliques were opened and fixed under vacuum in 4% paraformaldehyde, 0.5X MTSB (25 mM PIPES, 2.5 mM EGTA, 2.5 mM MgSO4, adjusted to pH 7 with KOH) and 0.1% triton for 1 hour. Samples were washed with 0.5 MTSB, 0.1% triton for 10 minutes and then transferred in Direct Red 23 (Sigma-Aldrich 212490, cas number 3441-14-3) 3 days, mounted in Citifluor/DAPI 20 mg/ml and observed with a confocal laser-scanning microscope [52].

#### Confocal microscopy and image acquisition

For mPS-PI, acquisitions were done with a Zeiss LSM 710 confocal microscope as described previously [53]. For cell wall and nucleus staining, excitation wavelength for DAPI was 405 nm and emission was collected from 410 to 460 nm. For Direct Red 23, the excitation wavelength was 561 nm and emission was collected from 565 to 720 nm. In all cases, fluorescence signals were recorded using a 40x objective and digitized as 8-bit 3D image stacks with a near-to-optimal voxel size of 0.17×0.17×0.35 *μ*m^3^.

Image acquisition of leaf epidermal cells was performed on a Leica SP 5II AOBS tandem HyD confocal laser-scanning microscope equipped with a Plan APOX NA1.25 oil immersion objective. GFP was excited at 488 nm by a diode laser and detected between 500 and 540 nm. RFP was excited at 561 nm and detected between 580 and 640 nm. Time-lapse images were acquired every 30 mn for several hours to detect cell divisions.

### Image processing and analysis

#### Image segmentation

A complete image-processing pipeline was designed to segment embryo cells in 3D confocal image stacks. Following image resampling to cubic voxels of side length 0.35 *μ*m, image denoising was performed thanks to adaptive filters [54]. A 2D crest line detector [55] was then applied across the sets of x-y, x-z, y-z slices and the results combined with the maximum operator. The 3D Euclidean distance map computed from the crest binary image was finally segmented using the watershed transform available in the Matlab Image Processing Toolbox (Copyright 1994-2013 The MathWorks, Inc).

#### Reconstruction of cell lineages automatic spatial annotation

Reconstructed embryos were composed of cells from the embryo proper and of few cells from the suspensor. Cell neighborhood analysis was used to automatically remove suspensor cells: starting with the cell with a single neighbor, cells were recursively removed until all remaining cells had at least two neighbors. Taking benefit from the stereotyped nature of cell division patterns during the first generations, cell divisions could be visually identified in the 3D segmented images based on geometrical cues and recursively rewound back to the 1C stage. Using the cell lineage, we could identify the first division plane *D*_1_ of the embryo proper. We then defined an embryo-centered coordinate frame $\vec{i},\vec{j},\vec{k}$ having its origin at the centroid *G*_1_ of *D*_1_ (S14 Fig): $\vec{i}$ was given by the normal to *D*_1_ and $\vec{k}$ by the projection of $\vec{G_{1}G_{2}}$ on *D*_1_, where *G*_2_ was the centroid of the last cell of the suspensor. The third vector was uniquely defined as $\vec{j}=\vec{i}\times \vec{k}$. Automatic spatial annotation of segmented cells and of mother cells reconstructed along lineage trees was then performed as follows. Based on projections of cell centroids on the $\vec{k}$ axis (S14 Fig), we first classified as central or apical the cells at the 8C stage. Propagating this classification along the lineage trees resulted in a central/apical annotation of cells in the subsequent generations. Projecting the centroids of these cells on the $\vec{i}$-axis then allowed their annotation as internal (close to the first division plane) or external (close to the external wall of the embryo).

#### Cell morphometric measurements

The centroid of each cell was computed and its size was quantified based on volume, wall surface area, mean breadth, lengths of the three main axes of the equivalent inertia ellipsoid, radius of the maximal enclosed ball, and geodesic diameter. These parameters were computed on the 3D cell masks, which were obtained either directly from the segmented images or following reconstruction of mother cells based on lineage trees. Several shape factors were computed for each cell: three shape factors computed as ratios of volume, surface area, and mean breadth; three elongation factors computed as ratios of ellipsoid axis lengths; convexity, computed as the ratio of volume over the volume of the convex hull; geodesic elongation, defined as the ratio of geodesic diameter over the diameter of the inscribed ball. In total, 16 morphometric features described each cell.

#### Division plane geometric measurements

For each division plane, a triangular mesh, a unit normal vector and a centroid were computed. The mesh was obtained by applying the *Generate Surface* function of Avizo Fire software (Copyright 2013 Visualization Sciences Group, an FEI Company) to the labeled image obtained by 3D segmentation. The unit normal vector was defined as the area-weighted average of normals to the triangular facets in the mesh. The centroid was defined as the projection of the center of inertia of the mesh on itself. Several parameters were then computed to characterize each division plane. The volume-ratio associated to each division plane was defined as the ratio between the volume of one daughter cell and the volume of the mother cell. Depending on the generation, the considered daughter cell was selected either as the smallest one (stages 2C, 4C, and 32C) or based on its spatial location in the embryo (apical/central at 8C, internal/external at 16C). The position of each division plane was quantified by the Euclidean distance between the mother cell centroid and the closest point on the triangular mesh representing the plane. The orientation of each division plane with respect to the external embryo wall was measured as the angle between its normal vector and the vector from *G*_1_ to the centroid of the mother cell. To quantify changes in division plane orientation between generations, the angle between normal vectors to division planes from successive generations was measured.

#### Statistical shape analysis

Using the individual cell morphometric features listed above, a multiple discriminant analysis was performed to quantitatively assess, at a given stage, the morphological differences between cell shapes in different domains of the embryo. The logarithm of the features was used to provide distributions closer to Gaussian ones. A linear discriminant analysis was applied on the centered and reduced data sets. We used the Fisher Linear Discriminant Analysis function contributed in Matlab by Sergios Petridis (https://fr.mathworks.com/matlabcentral/fileexchange/38950-fisher-linear-dicriminant-analysis). For 8C stage, the apical and central classes were used. For 16 and 32 cells, combinations of apical/central and internal/external classes were used. This resulted in transformed coordinates exhibiting the differences between the different cell classes.

#### Segmentation and measurements of dividing cells in leaf epidermis

The 2D binary masks of mother cells were obtained by running the watershed transform [28] on Gaussian filtered LTI6B-GFP images immediately prior to cell division. The 2D binary masks of the nuclei were obtained by intensity thresholding of the corresponding Gaussian filtered H2B-RFP images. Observed volume ratios were computed from the daughter cell masks obtained by running the watershed transform on the LTI6B-GFP images just after division.

### Computational model of cell division

A stochastic computational model of cell division in arbitrary 3D shapes was developed using a discrete-space approach along a formalism related to the cellular Potts model [43]. In our model, the mother cell space was divided into a finite number of sites (equivalent to voxels) and a division was represented as an assignment of each site to one of the two daughter cells (S6 Fig). To search for configurations satisfying volume-ratio and plane area constraints, an energy function was defined over the set of possible configurations {*x*}:

$$\begin{array}{c} H\left(x\right)=H_{V}\left(x\right)+H_{A}\left(x\right) \end{array}$$

The term *H*_*V*_(*x*) was defined to enforce the volume-ratio at a given value ρ*:

$$\begin{array}{c} \frac{H_{V}\left(x\right)}{kT}={\left({\left[V_{1}\left(x\right)-V_{1}^{*}\right]}^{2}+{\left[V_{2}\left(x\right)-V_{2}^{*}\right]}^{2}\right)}^{\frac{1}{3}} \end{array}$$

where *V*_1_(*x*) and *V*_2_(*x*) are the actual volumes of the daughter cells in configuration *x*, $V_{1}^{*}=\rho^{*}V$ and $V_{2}^{*}=\left(1-\rho^{*}\right)V$ are the desired volumes of the two daughter cells, with *V* being the volume of the mother cell. Setting ρ* = 0.5 would for example simulate symmetrical divisions (S6B Fig).

Since we based our initial investigations on the area minimization principle of Errera’s rule, the term *H*_*A*_(*x*) was defined to minimize the area of the division interface:

$$\begin{array}{c} \frac{H_{A}\left(x\right)}{kT}=\alpha \;\frac{a}{2}\sum_{(i,j)\text{neighbors}}1_{x[i]\neq x[j]} \end{array}$$

where $1_{x[i]\neq x[j]}$ is the indicator function of the event that neighbor sites *i* and *j* are assigned to a different daughter cell in configuration *x* and *a* is the area of a voxel facet, which was set from the original image spatial calibration. Estimating surface area by counting voxel facets can lead to surface orientation-dependent bias. Alternative methods include using the Crofton formula [56, 57] or 3D meshes interpolating through the 3D discrete grid. For the sake of computational efficiency, we defined here the neighborhood of a given voxel as the set of its 26 closest voxels, which is the equivalent of using the unweighted Crofton formula in global area estimation.

Configurations satisfying the volume-ratio and surface area constraints were obtained by stochastically minimizing *H* using the standard Metropolis algorithm [58]. Initial configurations were generated by randomly assigning each site of the mother cell to one of the two daughter cells (S6A Fig). These assignments were then iteratively modified by selecting, at each step, a random site and by computing the energy difference Δ*H* that would result from assigning this site to the other daughter cell. The new configuration was systematically accepted when Δ*H* ≤ 0 and was accepted with probability $\exp(-\frac{\Delta H}{kT})$ otherwise. A complete Monte-Carlo cycle was achieved by repeating this procedure *N* times, *N* being the number of sites in the mother cell binary mask. The algorithm was run for 5000 Monte-Carlo cycles, which, under our conditions, guaranteed that convergence had been reached. The model was run several times in each mother cell to explore the set of possible solutions.

All simulations were run with the same model (same constraints on volume-ratio and surface area) and parameter values, except for the target volume-ratio ρ*. Depending on the experiment, this parameter was set either to the observed value, to a value corresponding to a symmetric division (0.5) or to a value uniformly distributed at random over a large range of values in order to explore the space of possible solutions. The balance parameter *α* between the two terms in the energy function was fixed to 2 in all simulations. Setting *α* too low is tantamount to remove the surface area constraint, leading to a fragmented partitioning of the mother cell. Conversely, setting *α* too high gives too much emphasis to the surface area constraint and the obtained solutions can degenerate (one daughter cell invades the mother cell space because the formation of an interface is strongly penalized). The value 2 was empirically found in the early stages of the development of the model as a suitable compromise between noise and degeneracy. With this setup, the difference between the desired volume ratio ρ* and the actual volume ratio of the simulated divisions was below 1%.

For leaf epidermal cell divisions, the model was declined in a 2D version following the same principles. A simplification was introduced by removing from the energy function the term enforcing the volume-ratio constraint. Instead, the initial configurations were generated at the target volume-ratio ρ*. The volume-ratio was kept constant during the Metropolis procedure by performing site exchanges between the two daughter cells rather than toggling individual sites as in the 3D model.

The C++ code of the model and an executable version (Linux Ubuntu 16.04 64-bits) can be found at http://www-ijpb.versailles.inra.fr/en/bc/equipes/modelisation-imagerie/.

### Normalization of simulation results between cells

Division planes generated by the model were quantitatively analyzed by computing their surface area and their Euclidean distance to the centroid of the mother cell. Using a Marching Cubes algorithm [59], the division planes were extracted from the simulation 3D grid and were represented as triangular surface meshes on which these distance and area measurements were performed. To remove bias due to cell size differences, a normalization scheme was applied to allow pooling plane measurements from different mother cells. For each configuration *x* reproducing the experimentally observed plane (“concordant” solution), we computed its relative area $\hat{A}\left(x\right)$ comparatively to a set of alternative configurations S obtained on the same cell. This relative area was defined as the proportion of alternative configurations with a smaller area than *A*(*x*):

$$\begin{array}{c} \hat{A}\left(x\right)=\frac{1}{\left|S\right|}\sum_{x^{\prime }\in S}1_{A\left(x^{\prime }\right)<A\left(x\right)} \end{array}$$

By definition, the relative area is comprised between 0 and 1 (area smaller or larger, respectively, than all the alternative configurations); in addition, in case *x* is statistically equivalent to the alternative configurations, $\hat{A}\left(x\right)$ is uniformly distributed between 0 and 1. The same normalization procedure was used to compute a relative distance between division plane and cell centroid and, in the 2D case, to normalize division interface length and distance between division plane and nucleus centroid.

In some experiments, the set S of alternative configurations included simulation results that were obtained at the observed volume-ratio, as concordant solutions, but that were not reproducing the observed division pattern. In other experiments, S included simulation results obtained by drawing ρ* uniformly at random between 0.2 and 0.5.

### Visualization

Three-dimensional renderings of fluorescence intensity images, segmented images, and simulated divisions were achieved with the help of ImageJ/Fiji [60], Osirix [61], Avizo Fire software, and Free-D 3D viewer [62].

## Supporting information

S1 Fig Cell division patterns in *Arabidopsis thaliana* embryo development from 1C to 32C stages. (a-c) 1C stage with 3D volume rendering, longitudinal and radial sections; (d-f) 2C stage; (g-i) 4C stage; (j-m) 8C stage, (n-q) 16C-stage and (r-u) 32C stage with radial sections of the central and of the apical domains.
(TIF)

S2 Fig Invariant cell lineage in *Arabdopsis thaliana* early embryo. At generation G2 (right panel) embryos have two hemispherical cells H1 and H2, which lead to four cells Q1-4 at generation G3, that each divide in one central (C1-4) and one apical cell (A1-4). Cells at generation G4 lead to inner (I1-I8) and outer (O1-O8) cells at generation G5, which in turn give rise to thirty two cells at generation G6.
(TIF)

S3 Fig Diversity of cell shapes at the 32C stage. (a) Identical cell shapes in central outer cells resulting from a symmetric anticlinal division. (b) Different cell shapes in central inner cells resulting from a periclinal asymmetric division. (c) Different cell shapes in apical outer cells resulting from an anticlinal division. (d-e) Four different cell shapes in apical inner cells.
(TIF)

S4 Fig Frequency distribution of the cell division volume-ratio measured at the 32C stage. In each domain, the ratio was calculated between the smallest daughter cell and the mother cell volumes. Same color as in Fig 1A.
(TIF)

S5 Fig Correspondence between division planes and centroids of mother cells (*Yellow dots*) at successive embryo developmental stages. (a) 4C stage. (b) 8C stage. (c-d) 16C stage in central (c) and apical (d) domains. (e-h) 32C stage in central outer (e), central inner (f), apical outer (g), and apical inner (h) domains.
(TIF)

S6 Fig Discrete space approach for modeling cell divisions in 3D (2D illustration). The volume of the mother cell is discretized into a number of sites (voxels) assigned to one or the other daughter cell (*Yellow/Blue*). (A) Random initial configuration. (B) Energy levels associated to different configurations, for two values of the desired volume-ratio ρ*. The minimum energy levels are shown in blue. The right-most configuration is always penalized because of its high interface area. Depending on whether a symmetric or asymmetric division is simulated, the model will favor one of the two other configurations.
(TIF)

S7 Fig Simulated symmetric divisions within synthetic shapes. *Red*: interface between daughter cells at the end of simulations. (a) Sphere. (b) Half-sphere. (c) Quarter of sphere.
(TIF)

S8 Fig Simulated divisions at the 8C-16C stage transition: Relative area of simulated periclinal divisions obtained at ρ* = *ρ*_*obs*_ compared with simulated planes obtained at ρ* = 0.5. (TIF)

S9 Fig Simulated divisions at the 4C-8C transition: Concordant and alternative transverse divisions. The model produced two types of transverse divisions, with either the central (*Middle*) or the apical (*Right*) daughter cell as the largest cell. Only the first type reproduced the observed size polarity (*Left*) and passed through or near the cell centroid (*Red*).
(TIF)

S10 Fig Evaluation of the geometrical rule at the 1C-2C transition. (A) Distance to centroid as a function of surface of area of simulated division planes in a sample reconstructed mother cell from the 1C stage. (B-F) Relative distance to mother cell centroid (BC) and relative area (D-F) of simulated planes reproducing observed patterns in 5 mother cells. The normalized measures were obtained by comparison either with all alternative planes obtained at observed volume-ratios (*Blue*), with all planes obtained at random volume-ratios (*Orange*), or with planes passing as close to cell centroids among those obtained at random volume-ratios (*Green*).
(TIF)

S11 Fig Evaluation of the geometrical rule at the 2C-4C transition. (A) Distance to centroid as a function of surface of area of simulated division planes in a sample reconstructed mother cell from the 2C stage. (B-F) Relative distance to mother cell centroid (BC) and relative area (D-F) of simulated planes reproducing observed patterns in 5 mother cells. The normalized measures were obtained by comparison either with all alternative planes obtained at observed volume-ratios (*Blue*), with all planes obtained at random volume-ratios (*Orange*), or with planes passing as close to cell centroids among those obtained at random volume-ratios (*Green*). Numbers in parenthesis indicate heights of truncated histogram bars.
(TIF)

S12 Fig The proposed geometrical rule is valid at all generations: Comparison between observed and predicted division planes at 2, 4, 8, and 16C embryo stages. Computer simulations were run in mother cells (*Blue, transparent*) reconstructed by merging sister cells. The simulated divisions following the proposed geometrical rule (*Orange*) reproduced the observed patterns (*Green*).
(TIF)

S13 Fig Correspondence between cell centroid and nucleus position. (A) Volume rendering of DAPI-stained nuclei (*Green*) and Direct Red 23-stained walls (*Blue*) in a 7C embryo, with superimposed surface rendering of segmented cell walls (*Gray surfaces*). Yellow dots show the positions of cell centroids. (B) Distribution of measured nucleus-to-centroid distances in embryos from 2C–16C stages. (C) Surface of a central cell (*Gray*) and periclinal simulated plane (*Blue*) passing by the nucleus centroid (*Orange*). (DE) Comparison of periclinal simulated planes passing within 1 voxel distance of the nucleus centroid to alternative simulated planes (*n* = 5 cells, 500 simulations per cell): distribution of the relative distance to the nucleus centroid (D) and of the relative plane area (E).
(TIF)

S14 Fig Embryo coordinate frame. Origin *G*_1_ (*Red dot*) is positioned at the centroid of the first division plane *D*_1_ (*Yellow*). The first suspensor cell (*Pink*) with its centroid *G*_2_ (*Blue dot*) helps define the orientation of the *k*-axis. The *i*-axis is orthogonal to *D*_1_, while the *j*-axis completes the coordinate frame.
(TIF)
