# Supplementary figures and images for: Cell geometry determines symmetric and asymmetric division plane selection in Arabidopsis early embryos
Source: PLoS Comput Biol. 2019 Feb 11;15(2):e1006771. doi: 10.1371/journal.pcbi.1006771 (PMC6386405; doi:10.1371/journal.pcbi.1006771)

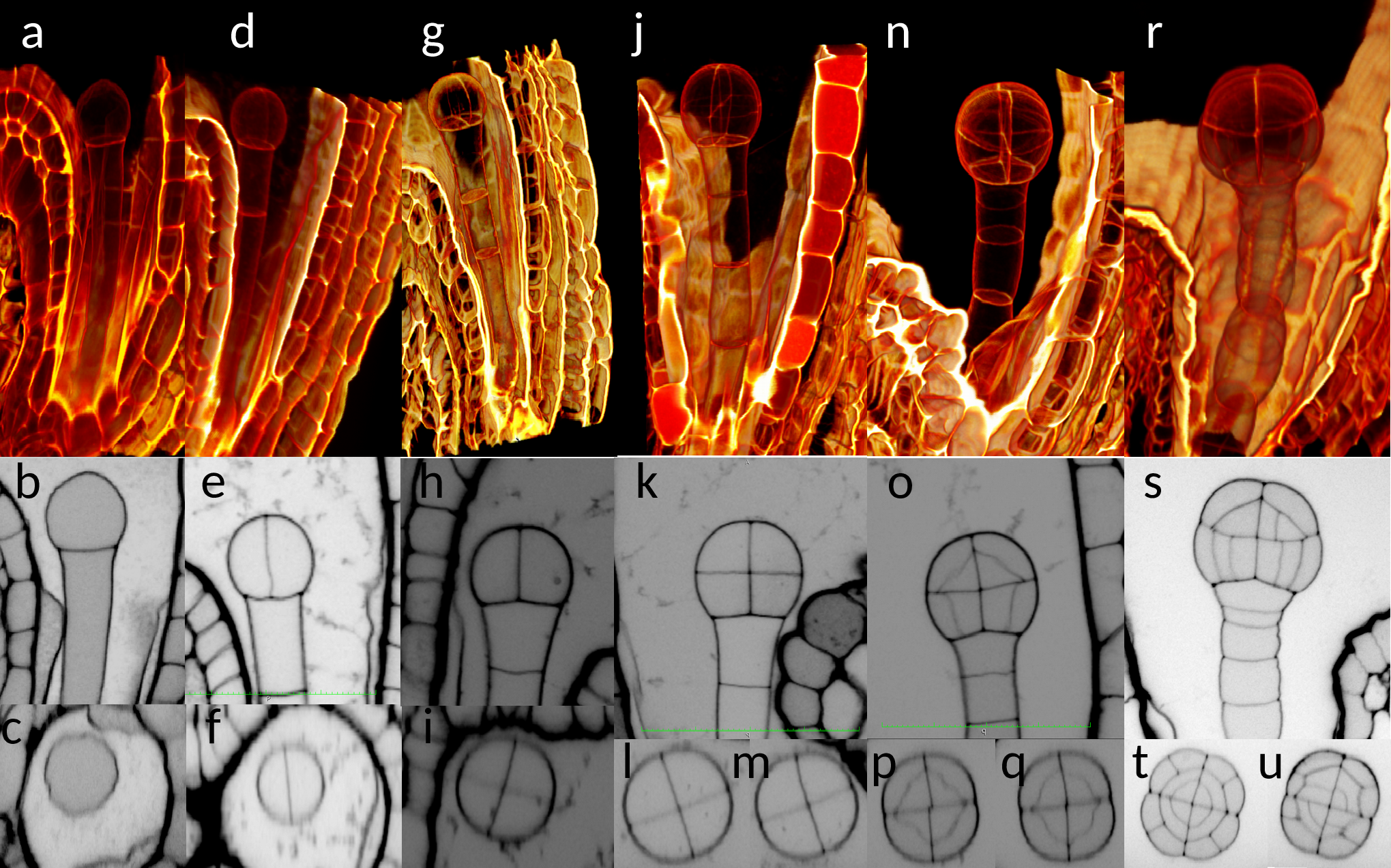

Supplement: S1 Fig — (a-c) 1C stage with 3D volume rendering, longitudinal and radial sections; (d-f) 2C stage; (g-i) 4C stage; (j-m) 8C stage, (n-q) 16C-stage and (r-u) 32C stage with radial sections of the central and of the apical domains. (TIF) [file pcbi.1006771.s001.tif]

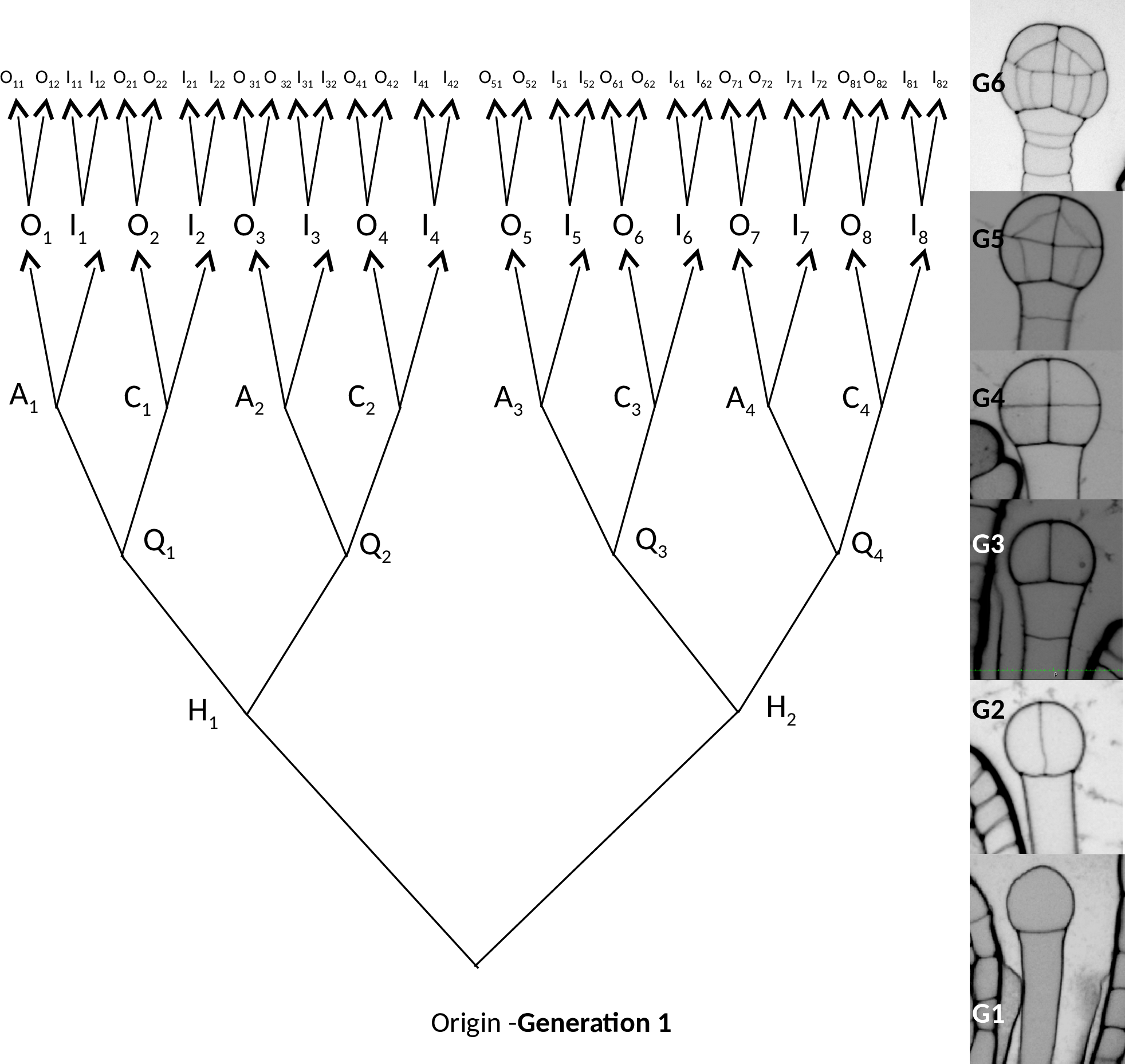

Supplement: S2 Fig — At generation G2 (right panel) embryos have two hemispherical cells H1 and H2, which lead to four cells Q1-4 at generation G3, that each divide in one central (C1-4) and one apical cell (A1-4). Cells at generation G4 lead to inner (I1-I8) and outer (O1-O8) cells at generation G5, which in turn give rise to thirty two cells at generation G6. (TIF) [file pcbi.1006771.s002.tif]

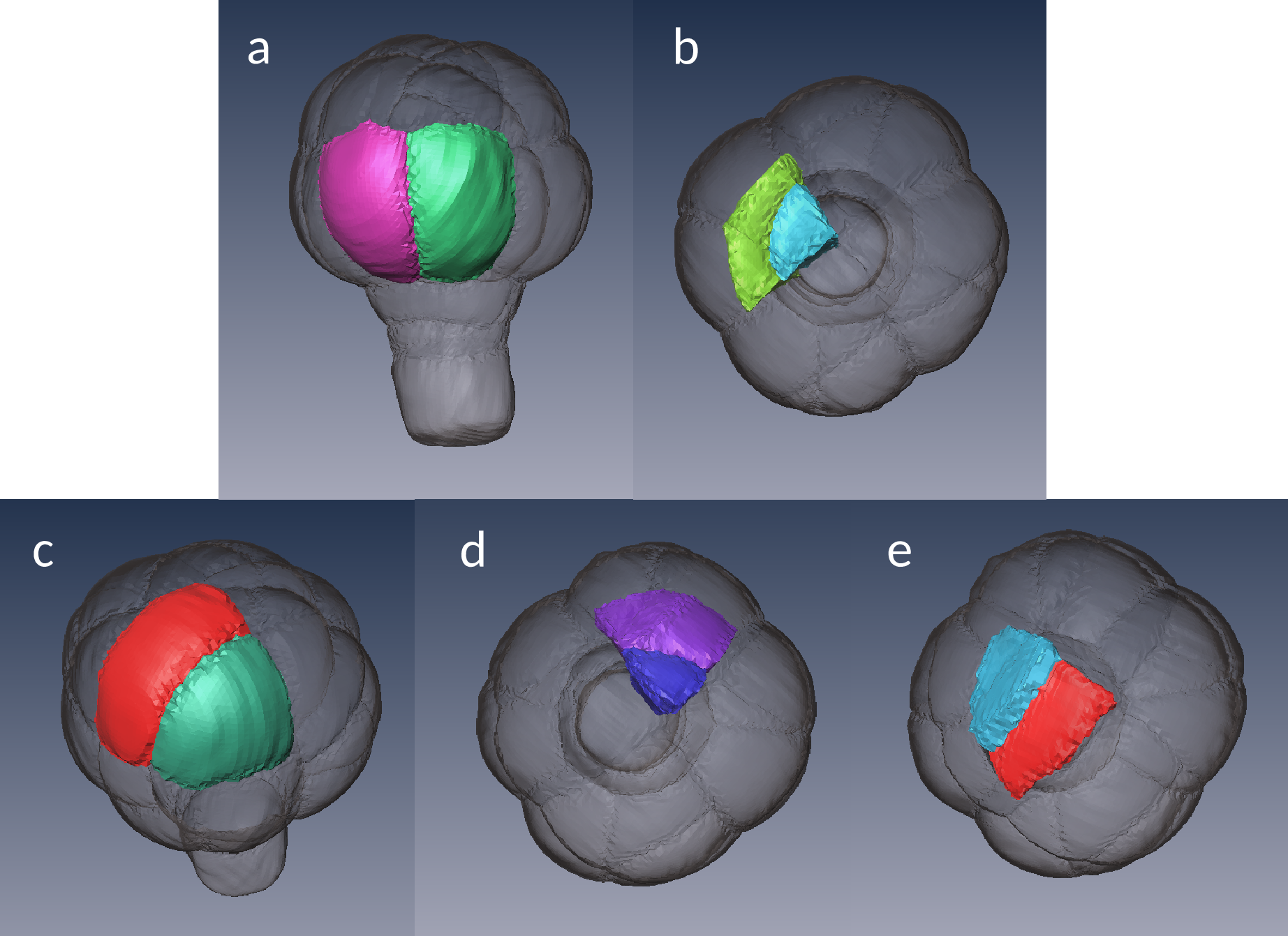

Supplement: S3 Fig — (a) Identical cell shapes in central outer cells resulting from a symmetric anticlinal division. (b) Different cell shapes in central inner cells resulting from a periclinal asymmetric division. (c) Different cell shapes in apical outer cells resulting from an anticlinal division. (d-e) Four different cell shapes in apical inner cells. (TIF) [file pcbi.1006771.s003.tif]

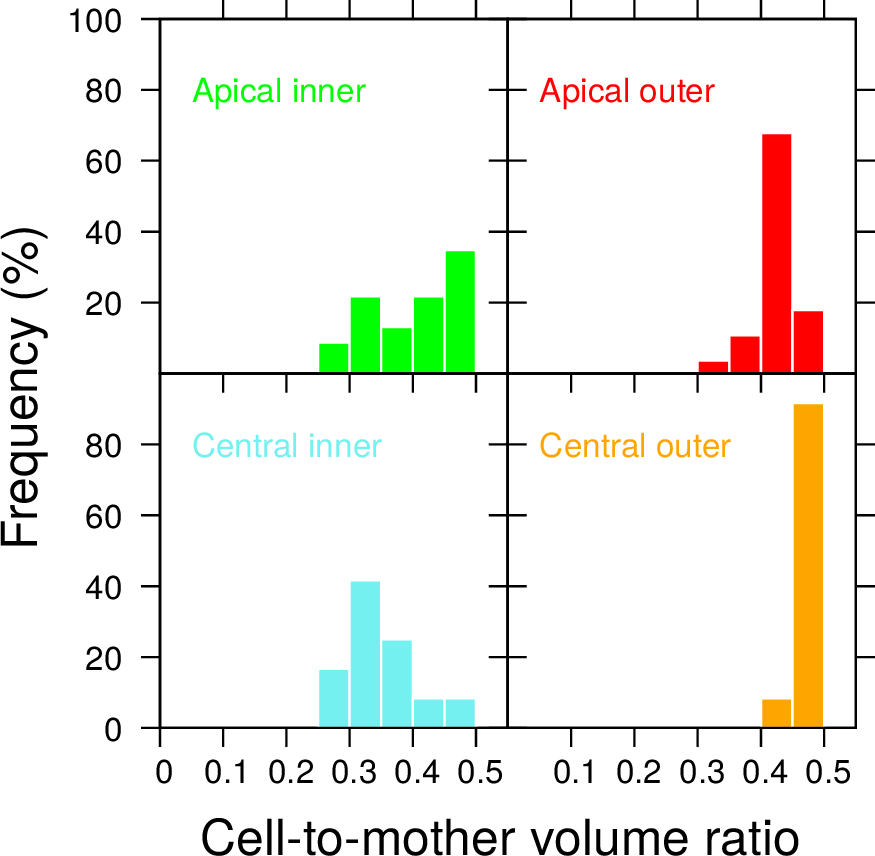

Supplement: S4 Fig — In each domain, the ratio was calculated between the smallest daughter cell and the mother cell volumes. Same color as in Fig 1A. (TIF) [file pcbi.1006771.s004.tif]

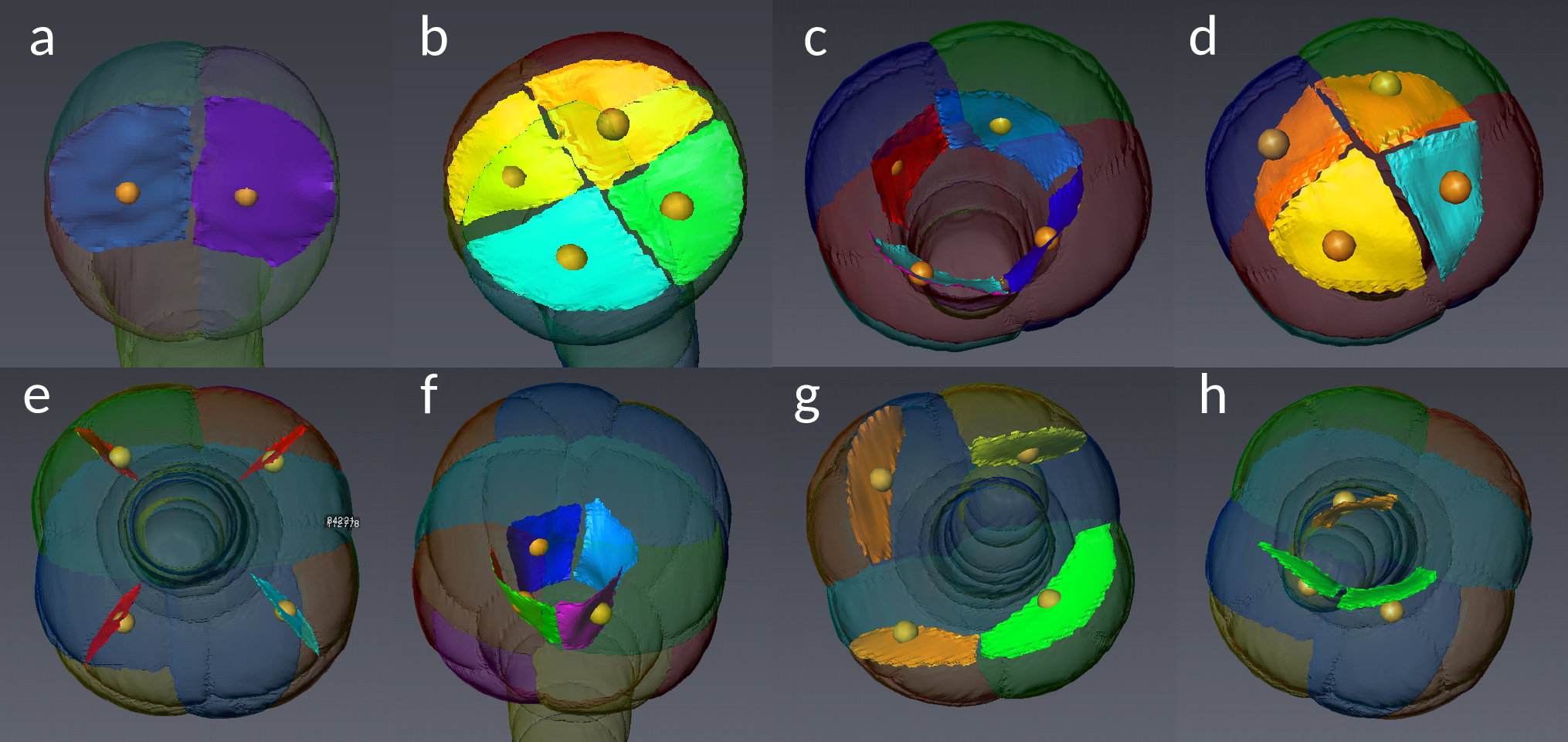

Supplement: S5 Fig — (a) 4C stage. (b) 8C stage. (c-d) 16C stage in central (c) and apical (d) domains. (e-h) 32C stage in central outer (e), central inner (f), apical outer (g), and apical inner (h) domains. (TIF) [file pcbi.1006771.s005.tif]

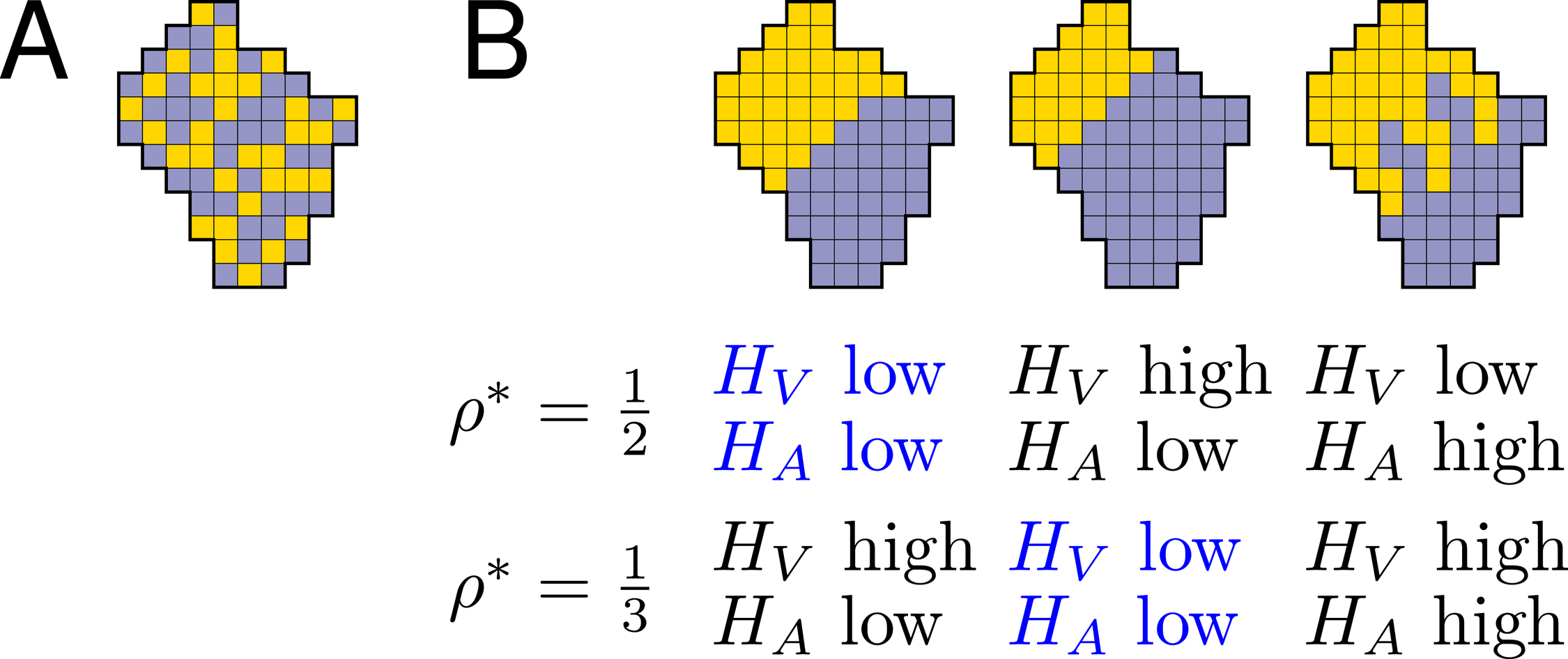

Supplement: S6 Fig — The volume of the mother cell is discretized into a number of sites (voxels) assigned to one or the other daughter cell (Yellow/Blue). (A) Random initial configuration. (B) Energy levels associated to different configurations, for two values of the desired volume-ratio ρ*. The minimum energy levels are shown in blue. The right-most configuration is always penalized because of its high interface area. Depending on whether a symmetric or asymmetric division is simulated, the model will favor one of the two other configurations. (TIF) [file pcbi.1006771.s006.tif]

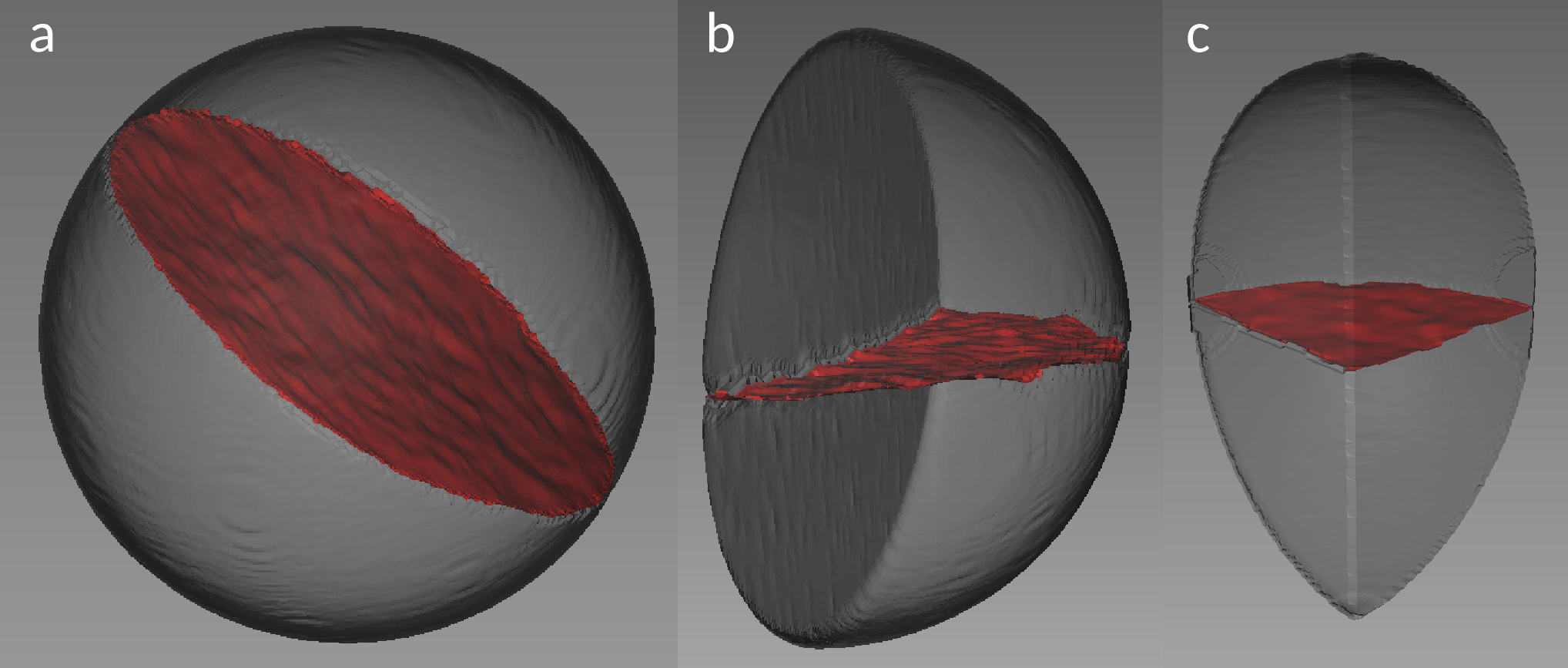

Supplement: S7 Fig — Red: interface between daughter cells at the end of simulations. (a) Sphere. (b) Half-sphere. (c) Quarter of sphere. (TIF) [file pcbi.1006771.s007.tif]

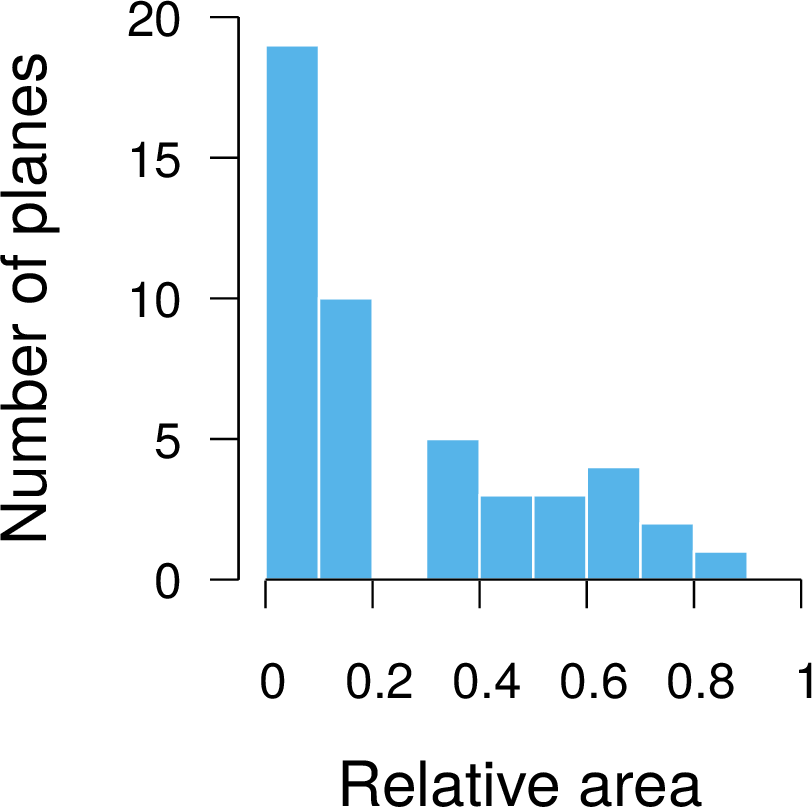

Supplement: S8 Fig — (TIF) [file pcbi.1006771.s008.tif]

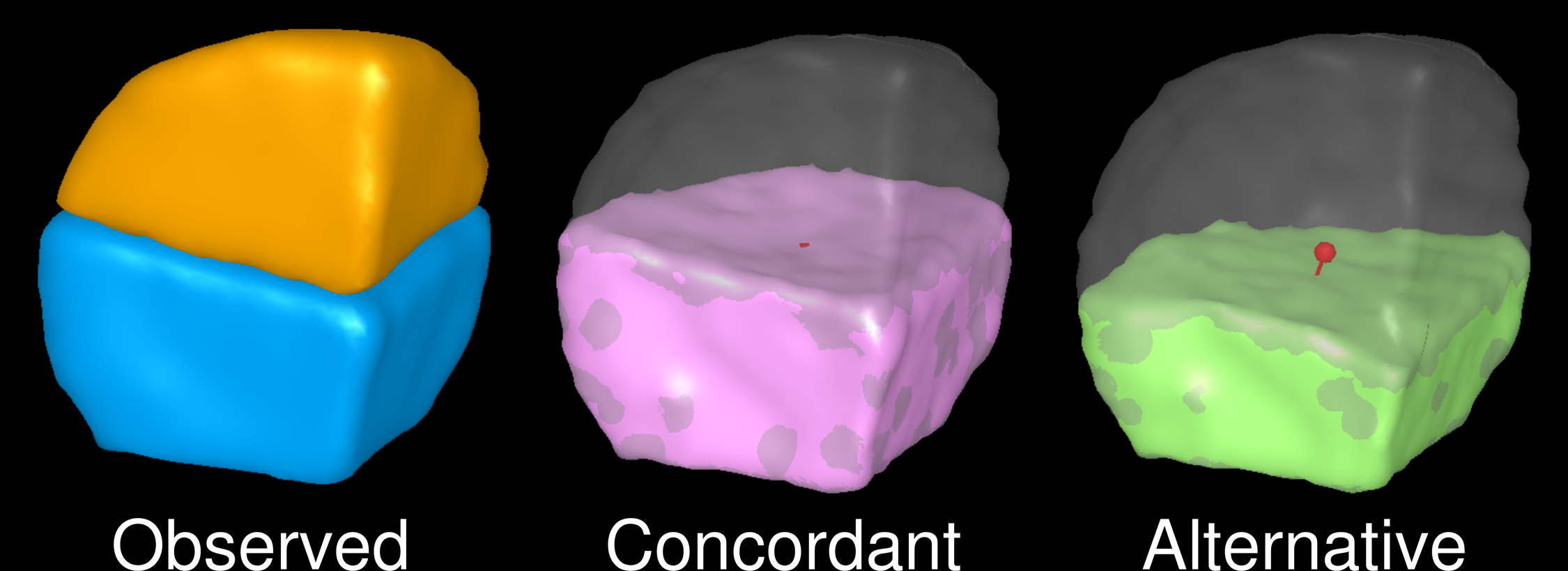

Supplement: S9 Fig — The model produced two types of transverse divisions, with either the central (Middle) or the apical (Right) daughter cell as the largest cell. Only the first type reproduced the observed size polarity (Left) and passed through or near the cell centroid (Red). (TIF) [file pcbi.1006771.s009.tif]

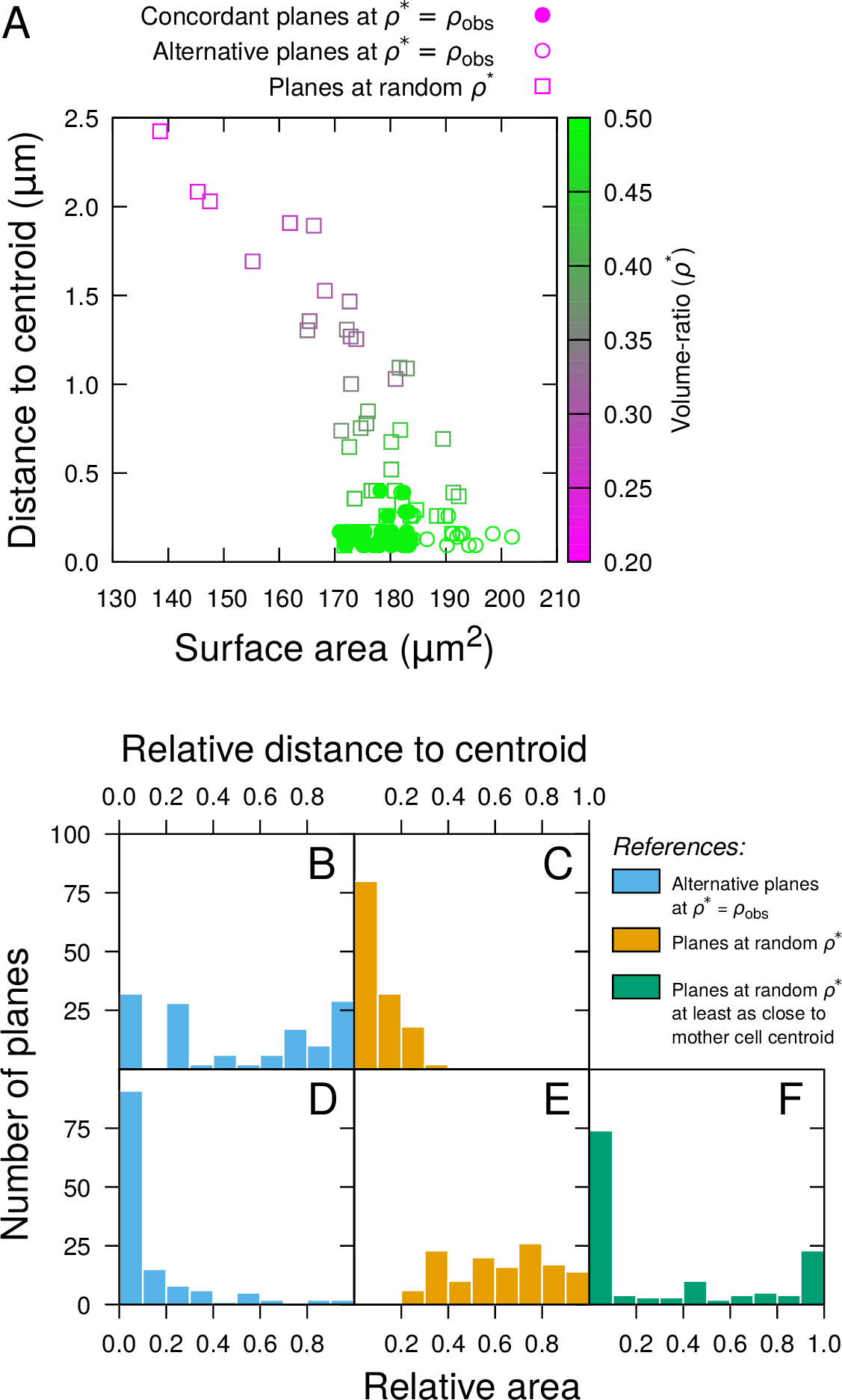

Supplement: S10 Fig — (A) Distance to centroid as a function of surface of area of simulated division planes in a sample reconstructed mother cell from the 1C stage. (B-F) Relative distance to mother cell centroid (BC) and relative area (D-F) of simulated planes reproducing observed patterns in 5 mother cells. The normalized measures were obtained by comparison either with all alternative planes obtained at observed volume-ratios (Blue), with all planes obtained at random volume-ratios (Orange), or with planes passing as close to cell centroids among those obtained at random volume-ratios (Green). (TIF) [file pcbi.1006771.s010.tif]

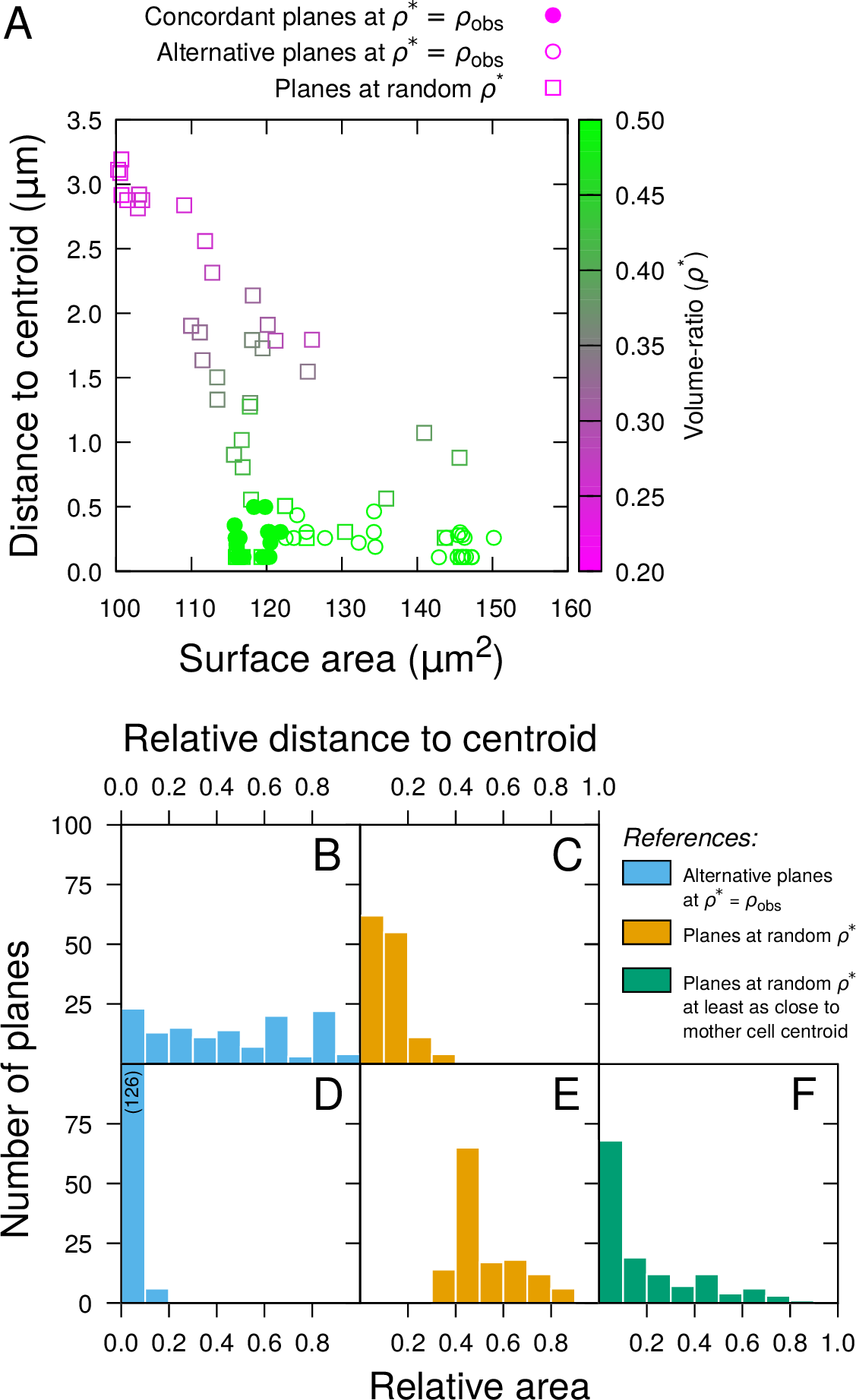

Supplement: S11 Fig — (A) Distance to centroid as a function of surface of area of simulated division planes in a sample reconstructed mother cell from the 2C stage. (B-F) Relative distance to mother cell centroid (BC) and relative area (D-F) of simulated planes reproducing observed patterns in 5 mother cells. The normalized measures were obtained by comparison either with all alternative planes obtained at observed volume-ratios (Blue), with all planes obtained at random volume-ratios (Orange), or with planes passing as close to cell centroids among those obtained at random volume-ratios (Green). Numbers in parenthesis indicate heights of truncated histogram bars. (TIF) [file pcbi.1006771.s011.tif]

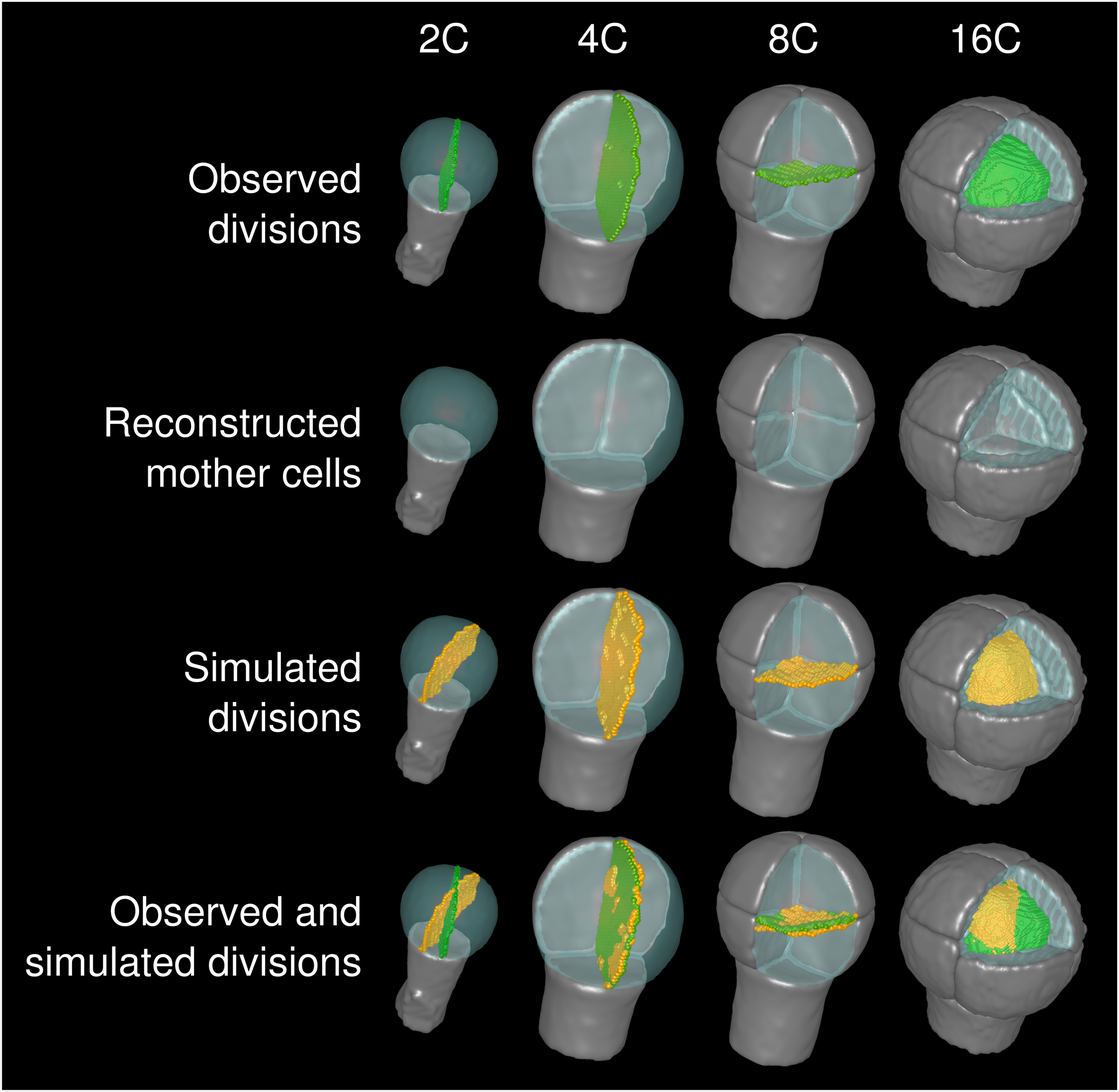

Supplement: S12 Fig — Computer simulations were run in mother cells (Blue, transparent) reconstructed by merging sister cells. The simulated divisions following the proposed geometrical rule (Orange) reproduced the observed patterns (Green). (TIF) [file pcbi.1006771.s012.tif]

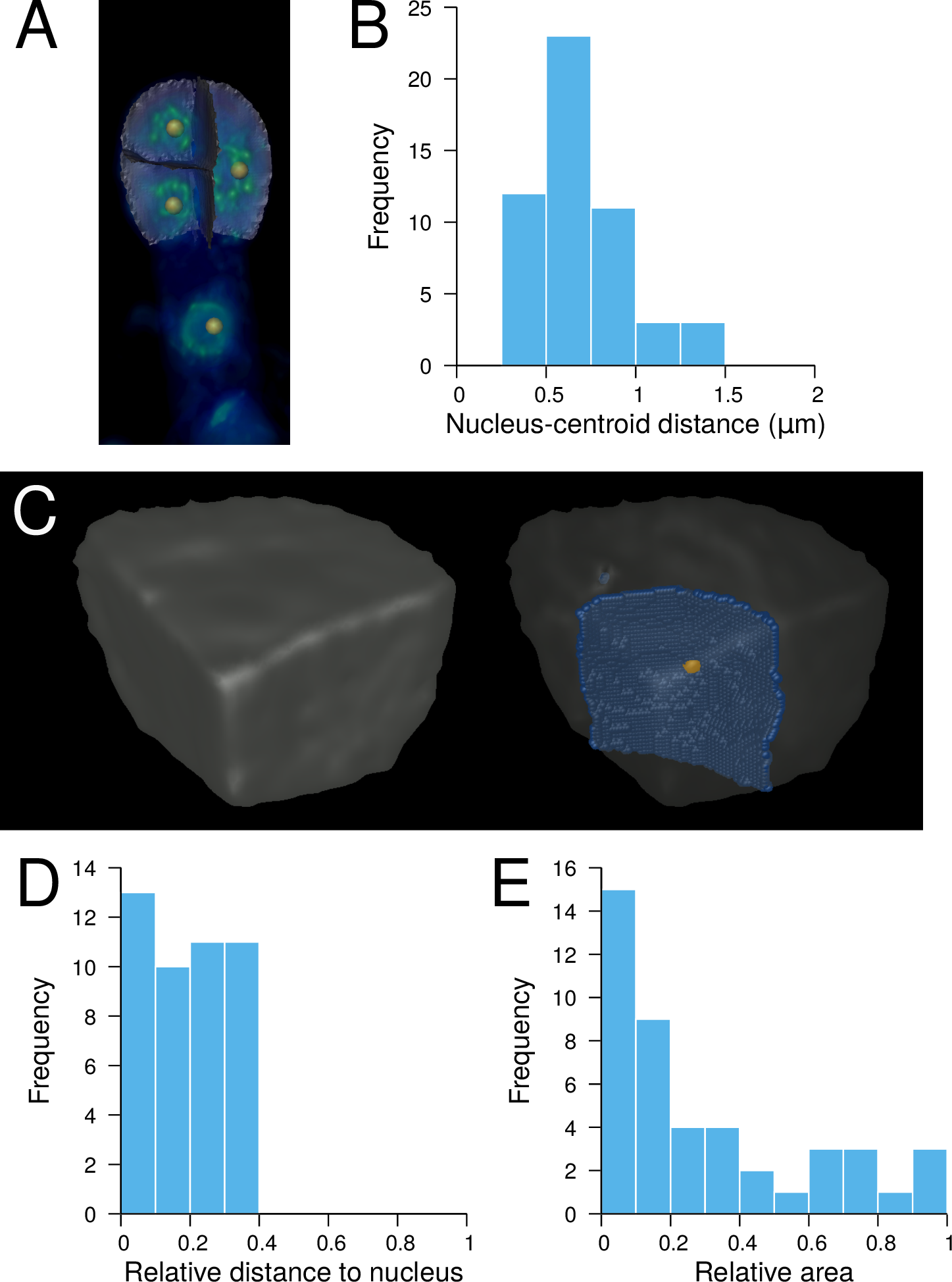

Supplement: S13 Fig — (A) Volume rendering of DAPI-stained nuclei (Green) and Direct Red 23-stained walls (Blue) in a 7C embryo, with superimposed surface rendering of segmented cell walls (Gray surfaces). Yellow dots show the positions of cell centroids. (B) Distribution of measured nucleus-to-centroid distances in embryos from 2C–16C stages. (C) Surface of a central cell (Gray) and periclinal simulated plane (Blue) passing by the nucleus centroid (Orange). (DE) Comparison of periclinal simulated planes passing within 1 voxel distance of the nucleus centroid to alternative simulated planes (n = 5 cells, 500 simulations per cell): distribution of the relative distance to the nucleus centroid (D) and of the relative plane area (E). (TIF) [file pcbi.1006771.s013.tif]

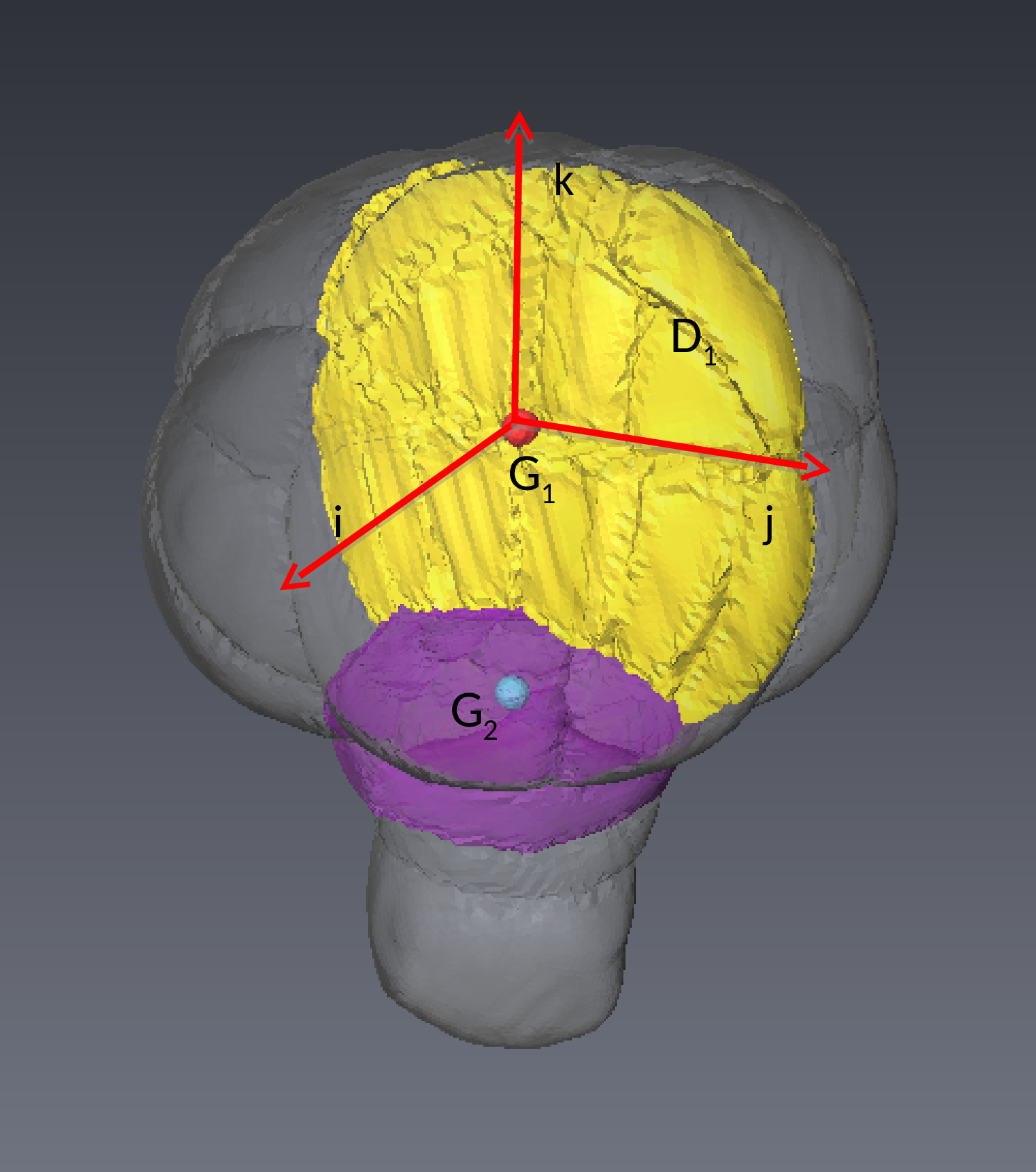

Supplement: S14 Fig — Origin G1 (Red dot) is positioned at the centroid of the first division plane D1 (Yellow). The first suspensor cell (Pink) with its centroid G2 (Blue dot) helps define the orientation of the k-axis. The i-axis is orthogonal to D1, while the j-axis completes the coordinate frame. (TIF) [file pcbi.1006771.s014.tif]
